# Computer-aided quality assurance versus standard colonoscopy: a systematic review and meta-analysis of randomized controlled trials

**DOI:** 10.1055/a-2904-8262

**Published:** 2026-08-06

**Authors:** Kristoffer Mazanti Cold, Martine S. Nielsen, Juana Maria Abdelreda, Flemming Bjerrum, Lars Konge

**Affiliations:** 1 Center for HR & Education 615551 CAMESCopenhagenDenmark; 2 Department of Clinical Medicine, Faculty of Health and Medical Sciences 4321 University of CopenhagenCopenhagenDenmark; 3 Department of Urology 53140 Zealand University Hospital RoskildeRoskildeDenmark; 4 Department of Anesthesiology and Intensive Care 371420 Lillebaelt Hospital - University Hospital of Southern DenmarkKoldingDenmark; 5 Research Unit of Medical Education 6174 University of Southern DenmarkOdenseDenmark; 6 GastroUnitCopenhagen University Hospital - Amager and HvidovreHvidovreDenmark

## Abstract

**Background:**

Computer-aided quality assurance (CAQ) can provide feedback to improve mucosal inspection during colonoscopy. This systematic review and meta-analysis of randomized controlled trials (RCTs) evaluated the efficacy of CAQ versus standard colonoscopy in enhancing colonoscopy quality indicators.

**Methods:**

Adhering to PRISMA guidelines, we searched MEDLINE, Embase, and CENTRAL for RCTs comparing CAQ with standard colonoscopy. The primary outcome was adenoma detection rate (ADR). Secondary outcomes included detection rates for advanced adenoma (AADR) and adenocarcinoma (ACDR), adenomas per colonoscopy (APC), and withdrawal time. Pooled risk ratios (RRs) and weighted mean differences (WMDs) were calculated using random-effects models.

**Results:**

Six RCTs involving 5687 colonoscopies (CAQ 2895; standard colonoscopy 2792) were included. Compared with standard colonoscopy, CAQ increased ADR by 43% (RR [95%CI]: 1.43 [1.17–1.73]; P < 0.001, I2 = 73.5%), with heterogeneity decreasing substantially after omitting one study (1.52 [1.32–1.75]; P < 0.001, I2 = 11.9%); AADR by 54% (1.54 [1.26–1.87]; P < 0.001, I2 = 0.0%); ACDR by 30% (1.30 [1.00–1.68]; P = 0.047, I2 = 0.0%); APC by 0.14 adenomas (WMD [95%CI]: 0.14 [0.08–0.20]; P < 0.001, I2 = 53.2%); adenomas ≥10 mm by 0.03 adenomas (0.03 [0.02–0.05]; P < 0.001, I2 = 45.1%); and withdrawal time increased by 0.64 minutes (0.64 [0.04–1.24]; P = 0.04, I2 = 96.1%).

**Conclusions:**

CAQ significantly improved ADR and detection of clinically significant lesions. While findings for AADR and ACDR should be interpreted with caution due to low event rates, the improvements in detection of adenomas ≥10 mm and APC suggest that CAQ may lower the risk of post-colonoscopy colorectal cancer, a hypothesis that warrants evaluation in future long-term analyses of patient outcomes.

## Abstract (de)

**Graphical abstract:**

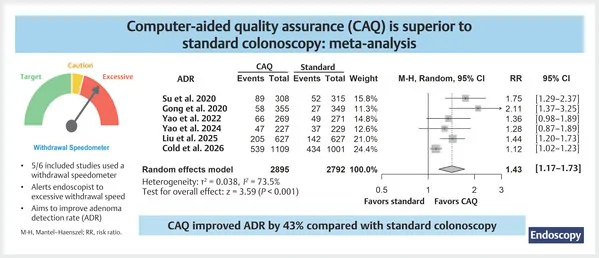

## Introduction

Colorectal cancer (CRC) is the **third most common cancer worldwide** and the **second leading cause of cancer-related** death 1 . In CRC, early detection and treatment through standard colonoscopy can substantially reduce mortality 2 , making high-quality endoscopic diagnosis and treatment central to CRC control strategies 3 . The adenoma detection rate (ADR) is inversely correlated to the post-colonoscopy CRC (PCCRC) rate 4 , and is the most widely used quality indicator reflecting endoscopists’ competence during colonoscopy 5 . Unfortunately, ADR varies widely among endoscopists, ranging from 8.2% to 68.1% 6 .

There are three reasons an adenoma might be missed, and these can explain the differences in ADR 7 ; artificial intelligence (AI) has provided systems to address each of them. First, a lesion could be visible during endoscopy but not recognized by the endoscopist. Several meta-analyses explored computer-aided polyp detection (CADe) based on AI and found that it improved the ADR 8 9 , but only for smaller adenomas, which might not lower PCCRC 10 . Second, a lesion could be obscured, for example by stool, fluids, or other structures. Computer-aided assessment of bowel preparation has been correlated to ADR 11 , but no studies have shown that using the system could help to improve ADR 12 . Finally, a lesion could be located in a mucosal area that was not visualized during the colonoscopy. Several computer-aided quality assurance (CAQ) feedback systems have been developed: for example, a withdrawal speedometer that warns the endoscopist that they are withdrawing too quickly, a system that assesses fold examination quality, and a scope movement analysis system that provides the endoscopist with feedback on the thoroughness of their inspection 13 .

CAQ is the only system that directly addresses the inspection quality 14 ; CAQ potentially provides a better AI system than CADe for improving an endoscopist’s competence and might be superior to CADe in improving ADR 15 . CAQ studies have either correlated their scores to ADR or shown ADR improvement by providing feedback. However, a systematic review found results to be inconsistent 13 , and no meta-analysis has been conducted to explore whether CAQ systems are superior to standard colonoscopy in improving colonoscopy performance in randomized controlled trials (RCTs).

This systematic review and meta-analysis aimed to compare CAQ with standard colonoscopy in RCTs.

## Methods

The systematic review adhered to the Preferred Reporting Items for Systematic Reviews and Meta-Analyses (PRISMA) guidelines 16 (see **Table 1s** in the online-only Supplementary material) and was registered in the International Prospective Register of Systematic Reviews (PROSPERO, registration number CRD-420261338110).

The systematic literature review was performed by searching MEDLINE (via PubMed), Embase, and CENTRAL. The search string used Boolean operators by using key elements of the research question for PubMed; it was then adapted for the other databases. A research librarian from the University of Copenhagen assisted in adapting the search string from a previous systematic review on CAQ systems in colonoscopy 13 , including new studies but limiting the search to RCTs. The search excluded conference abstracts and was limited to English language (see **Supplementary methods** , **Fig. 1s** , and **Fig. 2s** for search strings). The period spanned from 2020 to the present, as several systematic reviews did not identify any CAQ RCTs published prior to 2020.

Covidence systematic review software (Veritas Health Innovation, Melbourne, Australia) removed duplicates, and was used for title and abstract screening, full-text screening, and data extraction by two authors (K.M.C. and M.S.N.) independently. Any discrepancies were resolved through consensus. If consensus could not be reached between the two authors, a third author (L.K.) made the final decision.

### Eligibility criteria

All original RCTs investigating CAQ versus standard colonoscopy of single colonoscopy procedures in a clinical setting were included. Inclusion criteria were RCTs in a clinical setting reporting on CAQ systems that could provide immediate intra-procedure or post-procedure feedback on single colonoscopy performance. Exclusion criteria were CAQ systems monitoring quarterly ADR, cecal intubation rate etc., but not providing instant feedback on single procedures, or RCTs that only evaluated lesions of visualized mucosa (CADe).

Forward and backward citation search was conducted using **Web of Science Core Collection (Clarivate Analytics, Philadelphia, Pennsylvania, USA)** to identify additional relevant studies, examining both the references cited by included articles and subsequent publications citing them.

### Data extraction

Data extracted from the included studies were divided into the following parts.

1. Description of the CAQ feedback system: the identified studies were grouped into the same category if they used identical CAQ feedback systems.

2. General study characteristics: year of publication, study design, number of centers, country, indication for colonoscopies, number of colonoscopies, patients (age and sex), endoscopists (number and description), definitions of withdrawal time, advanced adenomas, and adverse events.

3. Outcome measures: the primary outcome was ADR. Secondary outcome were other quality indicators that have been linked to PCCRC. The following dichotomous outcome measures were included: detection rates for advanced adenomas (AADR), adenocarcinomas (ACDR), and polyps (PDR), and adverse events. The following continuous and interval outcome measures were included: number of adenomas per colonoscopy (APC), number of polyps per colonoscopy (PPC), withdrawal time, adenoma size (≤5 mm, 6–9 mm, and ≥10 mm), and adenoma location (cecum, ascending colon, transverse colon, descending colon, sigmoid colon, and rectum). Data were extracted according to Cochrane guidelines from intention-to-treat rather than per-protocol analysis.

4. Risk of bias assessment: this was conducted using the revised Cochrane Risk of Bias Tool (RoB 2) for randomized studies.

Missing data were obtained upon request from existing systematic reviews, or by appropriate statistical methods.

### Statistical analysis

For dichotomous outcomes, pooled effect estimates were calculated as risk ratios (RRs) with corresponding 95%CIs using the Mantel–Haenszel method. For continuous outcomes, pooled estimates were calculated as weighted mean differences (WMDs) with 95%CIs utilizing the inverse variance method. To account for anticipated clinical and methodological variability across the included trials, all meta-analyses were conducted using a random-effects model, employing the DerSimonian–Laird estimator to calculate between-study variance *τ ^2^* 17 . Forest plots were generated to visualize individual study estimates and pooled effects.

Statistical heterogeneity among the studies was assessed and quantified using the Higgins *I ^2^* statistic 18 . To assess the robustness of the pooled results and evaluate the influence of individual trials on both the effect size and heterogeneity, a leave-one-out sensitivity analysis was performed. This analysis was not conducted for outcomes demonstrating an *I ^2^* of 0.0%, as the observed variance across studies did not exceed the variance expected by chance. An *I ^2^* = 0.0% was anticipated for certain outcomes, consistent with early CADe meta-analyses limited by a low number of included studies and low event rates 9 . Furthermore, a secondary sensitivity analysis was performed grouping the included studies into the utilized CAQ system category, in order to isolate the independent effect of each category.

All statistical analyses were conducted using the *meta* package in R, version 4.4.1 (R Foundation for Statistical Computing, Vienna, Austria), and results were considered statistically significant at a two-sided *P* value of <0.05.

## Results

The search was conducted on 8 March 2026 and identified 1064 studies after removal of duplicates. Six RCTs passed the eligibility criteria ( **Fig. 3s** ).

### Study characteristics

The six RCTs used three different CAQ systems: ENDOANGEL 15 19 20 , Automatic Quality Control System (AQCS) 21 22 , and Copenhagen Colonoscopy Concept 23 ( **Table 2s** ). Five out of the six studies used a withdrawal speedometer, and the remaining study used immediate and cumulative post-colonoscopy feedback through scope movement analysis 23 . Notably, the two studies using AQCS also entailed a CADe function 21 22 .

The RCTs included 5687 colonoscopies (CAQ 2895 colonoscopies; standard colonoscopy 2792 colonoscopies) by 60 endoscopists (weighted mean: 95 colonoscopies per endoscopist) ( Table 1 ). The weighted mean patient age was 55.3 vs. 55.2 years, and weighted mean female sex was 46.9% vs. 48.2% in the CAQ vs. standard colonoscopy groups, respectively. The studies were performed in China (five studies) and Denmark (one study). Three RCTs were multicenter 20 22 23 and three were single-center studies 15 19 21 . One RCT was a three-group tandem study, with the adenoma miss rate as its primary outcome and the first-pass detection measures included in the analyses 20 . The five remaining studies had ADR as their primary outcome. One RCT used four groups (standard colonoscopy, CAQ, CADe, CADe+CAQ) 15 , with CAQ and standard colonoscopy included in the current analyses, while the remaining five studies contained two groups (CAQ vs. standard colonoscopy).

**Table:** **Table 1** General study and patient characteristics.

Study, year [ref.] Country	Study design No. of centers	Indication	Patient age , years Female sex, %	Colonoscopies	Endoscopists, n Description	Definitions
Su et al. 2020 21 China	Parallel (2 groups) 1	Screening 35% Surveillance 0% Symptoms 65% Fit+ 0%	51.1 48.6%	CAQ 308 Standard 315 Total 623	6 4–6 years’ experience and colonoscopy volume 5000–8000	Withdrawal time: excluding biopsy time Advanced adenoma: NA Adverse events: NA
Gong et al. 2020 19 China	Parallel (2 groups) 1	Screening 17% Surveillance 5% Symptoms 77% Fit+ 0%	49.5 47.2%	CAQ 355 Standard 349 Total 704	6 1–3 years’ endoscopy experience and a total colonoscopy volume of 1500–4000	Withdrawal time: excluding biopsy or treatment time Advanced adenoma: NA Adverse events: NA
Yao et al. 2022 15 China	Parallel (4 groups ^1^ ) 1	Screening 89% Surveillance 10% Symptoms 1% Fit+ 0%	50.4 53.1%	CAQ 269 Standard 271 Total 540	8 NA	Withdrawal time: total withdrawal time Advanced adenoma: adenomas ≥10 mm diameter, HGD, or villous histology Adverse events: NA
Yao et al. 2024 20 China	Tandem (3 groups ^2^ ) 3	Screening 64% Surveillance 8% Symptoms 28% Fit+ 0%	50.2 48.3%	CAQ 227 Standard 229 Total 456	8 Novices (20 colonoscopies) only performed the retraction; experienced endoscopist (>5000 colonoscopies) performed the insertion to the cecum	Withdrawal time: excluding therapeutic interventions and washing Advanced adenoma: adenomas ≥10 mm diameter, HGD, and villous histology Adverse events: NA
Liu et al. 2025 22 China	Parallel (2 groups) 6	Screening 33% Surveillance 8% Symptoms 59% Fit+ 0%	51.2 48.4%	CAQ 627 Standard 627 Total 1254	15 >1500 colonoscopies	Withdrawal time: total withdrawal time without interventions Advanced adenoma: NA Adverse events: NA
Cold et al. 2026 23 Denmark	Parallel (2 groups ^3^ ) 3	Screening 0% Surveillance 0% Symptoms 0% Fit+ 100%	63.2 43.9%	CAQ 1109 Standard 1001 Total 2110	17 Experienced doctors and nurses (>300 colonoscopies annually)	Withdrawal time: total withdrawal time Advanced adenoma: adenomas ≥10 mm diameter, HGD, and villous histology Adverse events: uncontrollable bleeding or perforation. One patient suffered from severe bradycardia, which was initially treated as cardiac arrest
Total (mean) or weighted mean	NA 15 (2.5)	Screening 27% Surveillance 4% Symptoms 32% Fit+ 37%	55.3 46.9%	CAQ 2895 (483) Standard 2792 (465) Total 5687 (948)	60 (12.5) NA	Withdrawal time: NA Advanced adenoma: NA Adverse events: NA
CADe, computer-aided detection; CAQ, computer-aided quality assurance; FIT+, positive fecal immunochemical test; HGD, high grade dysplasia; NA, not available. ^1^ Standard colonoscopy, CAQ, CADe, and CADe+CAQ arms. ^2^ Tandem with initial colonoscopy by novice and second by experienced endoscopist. ^3^ Cluster randomized.						

The indication for colonoscopy varied significantly across the studies (weighted mean: screening 27%, surveillance 4%, symptoms 32%, and positive fecal immunochemical test 37%; chi-squared *P* < 0.001).

### Risk of bias assessment

Five out of six included RCTs had a low risk of bias for all domains. In the Cold et al. study 23 , there were some concerns about the randomization process and deviations from the intended interventions ( Fig. 1 ).

**Fig. 1:**
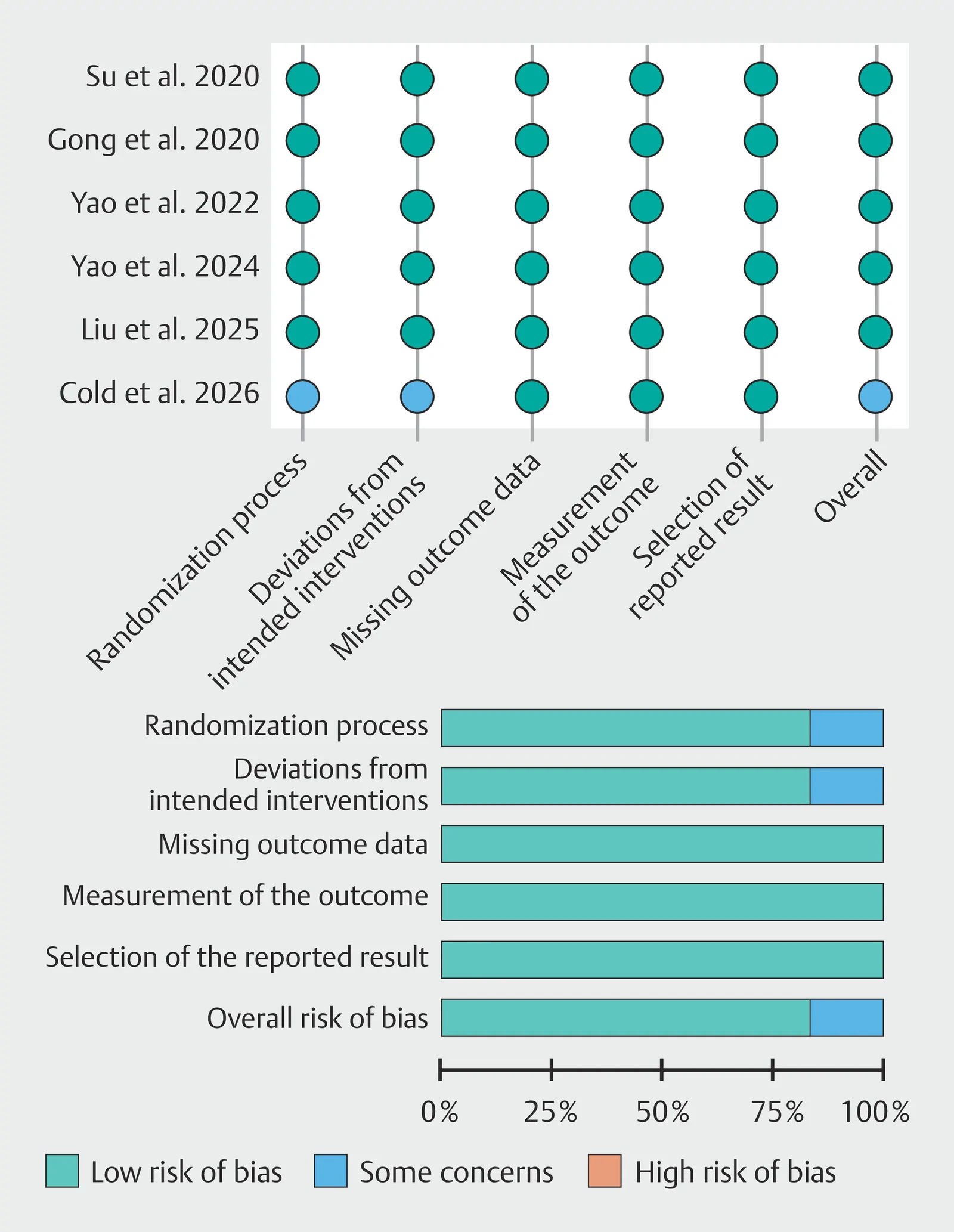
Risk of bias charts. Five out of six randomized controlled trials (RCTs) demonstrated a low risk of bias across all domains according to the RoB 2 tool – an identical rating by another systematic review for the four oldest RCTs 8 . There were “some concerns” about Cold et al. regarding the randomization process and deviations from intended interventions. This was due to its stepped-wedge cluster-randomized design with only three clusters, and technical difficulties with the computer-aided quality assurance system that prolonged the control period.

### ADR

Compared with standard colonoscopy, the use of CAQ increased ADR by 43% (RR 1.43 [95%CI 1.17–1.73]; absolute difference +8.1%; *P* < 0.001, *I ^2^* = 73.5%), with heterogeneity being considerably reduced by omitting Cold et al. 23 (RR 1.52 [95%CI 1.32–1.75]; absolute difference 8.9%; *P* < 0.001, *I ^2^* = 11.9%) ( Table 2 , Fig. 2 , **Fig. 4s** ). The increase in ADR remained significant with omission of each study.

**Table:** **Table 2** Summary of meta-analysis outcomes comparing computer-aided quality assurance vs. standard colonoscopy. The table presents the pooled effect estimates (risk ratios) alongside their 95%CIs, calculated using a random-effects model. Due to the high statistical heterogeneity observed across the primary analyses, a leave-one-out sensitivity analysis was conducted, omitting the study that decreased the *I ^2^* value the most.

	Events, n/N ^1^ (%)		RR [95%CI] (absolute difference) ^2^	P value ^2^	I ^2^ , %	τ ^2^
	CAQ	Standard colonoscopy				
ADR	1004/2895 (34.7)	741/2792 (26.5)	1.43 [1.17–1.73] (+8.1%)	<0.001	73.5	0.038
ADR leave-one-out ^3^	465/1786 (26.0)	307/1791 (17.1)	1.52 [1.32–1.75] (+8.9%)	<0.001	11.9	0.030
AADR ^4,5,6^	237/2232 (10.6)	143/2128 (6.7)	1.54 [1.26–1.87] (+3.9%)	<0.001	0	0
ACDR ^4,5^	131/2603 (5.0)	93/2488 (3.7)	1.30 [1.00–1.68] (+1.3%)	0.047	0	0
PDR ^7^	1162/2268 (51.2)	936/2165 (43.2)	1.24 [1.09–1.42] (+8.0%)	0.001	70.5	0.015
PDR leave-one-out ^3^	561/1786 (31.4)	428/1791 (23.9)	1.30 [1.16–1.46] (+7.5%)	<0.001	28.9	0.004
Adverse events ^4,5,8^	2/2360 (0.1)	5/2248 (0.2)	0.40 [0.07–2.23] (–0.1%)	0.30	0	0
AADR, advanced adenoma detection rate; ACDR, adenocarcinoma detection rate; ADR, adenoma detection rate; CAQ, computer-aided quality assurance; PDR, polyp detection rate; RR, risk ratio. ^1^ n = number detected; N = total number of colonoscopies. ^2^ Random-effects model. ^3^ Omitting Cold et al. 2026 23 . ^4^ Leave-one-out analysis not applied as * I ^2^* = 0.0% for the original analysis. ^5^ Data not available from Su et al. 2020 21 or left out due to leave-one-out sensitivity analysis. ^6^ Omitting Gong et al. 2020 19 . ^7^ Omitting Liu et al. 2025 22 . ^8^ Omitting Yao et al. 2024 20 .						

**Fig. 2:**
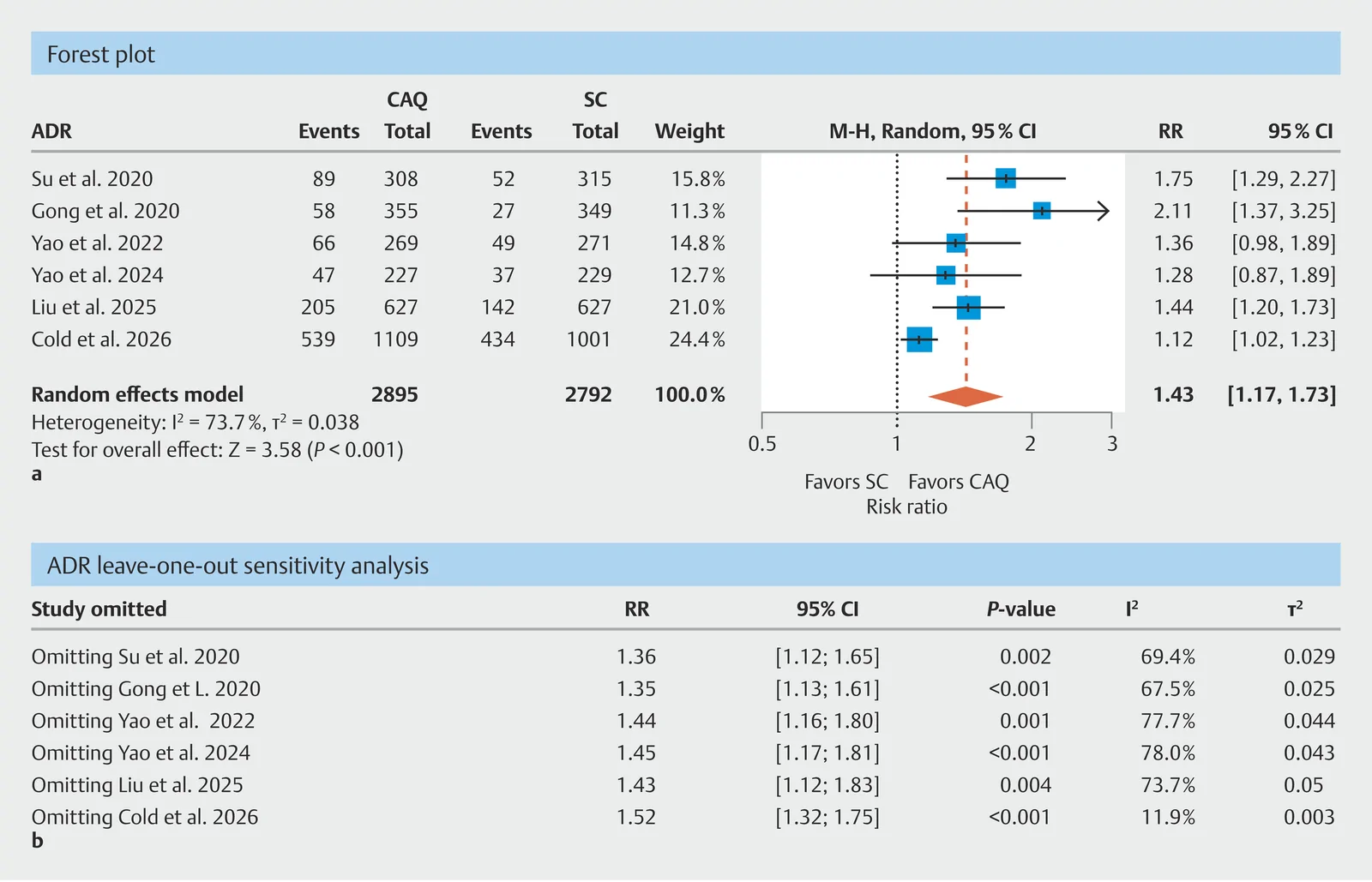
Forest plot and leave-one-out sensitivity analysis of adenoma detection rate (ADR). **a** Forest plot comparing ADR between computer-aided quality assurance (CAQ) intervention and standard colonoscopy. The overall effect indicated that the use of CAQ significantly increased ADR by 43% compared with standard colonoscopy (risk ratio [RR] 1.43 [95%CI 1.17–1.73]; *P* < 0.001). Given the substantial heterogeneity observed in this primary analysis ( *I ^2^* = 73.5%), a leave-one-out sensitivity analysis was conducted to identify sources of variance from individual studies. **b** Leave-one-out sensitivity analysis. Each row demonstrates the recalculated metrics when the specified study was omitted from the meta-analysis. Noticeably, omitting Cold et al. reduced the heterogeneity remarkably (from *I ^2^* = 73.5% to *I ^2^* = 11.9%). Omission of a single study kept resulting in a significant RR (range RR 1.35–1.52) favoring CAQ over standard colonoscopy. M-H, Mantel–Haenszel; SC, standard colonoscopy.

### Dichotomous secondary outcome measures (AADR, ACDR, PDR, and adverse events)

Compared with standard colonoscopy, CAQ increased AADR by 54% (RR 1.54 [95%CI 1.26–1.87]; absolute difference +3.9%; *P* < 0.001, *I ^2^* = 0.0%) ( Table 2 , Fig. 3 , **Fig. 5s** ). For ACDR the increase was 30% (RR 1.30 [95%CI 1.00–1.68]; absolute difference +1.3%; *P* = 0.047, *I ^2^* = 0.0%) ( **Fig. 6s** ). For PDR the increase was 24% (RR 1.24 [95%CI 1.09–1.42]; absolute difference +8.0%; *P* = 0.001, *I ^2^* = 70.5%), and with omission of Cold et al. 23 increased to 30% (RR 1.30 [95%CI 1.16–1.46]; absolute difference +7.5%; *P* < 0.001, *I ^2^* = 28.9%) ( **Fig. 7s** ). For adverse events, the reduction was 60% (RR 0.40 [95%CI 0.07–2.23]; absolute difference –0.1%); *P* = 0.30, *I ^2^* = 0.0%) ( **Fig. 8s** ).

**Fig. 3:**
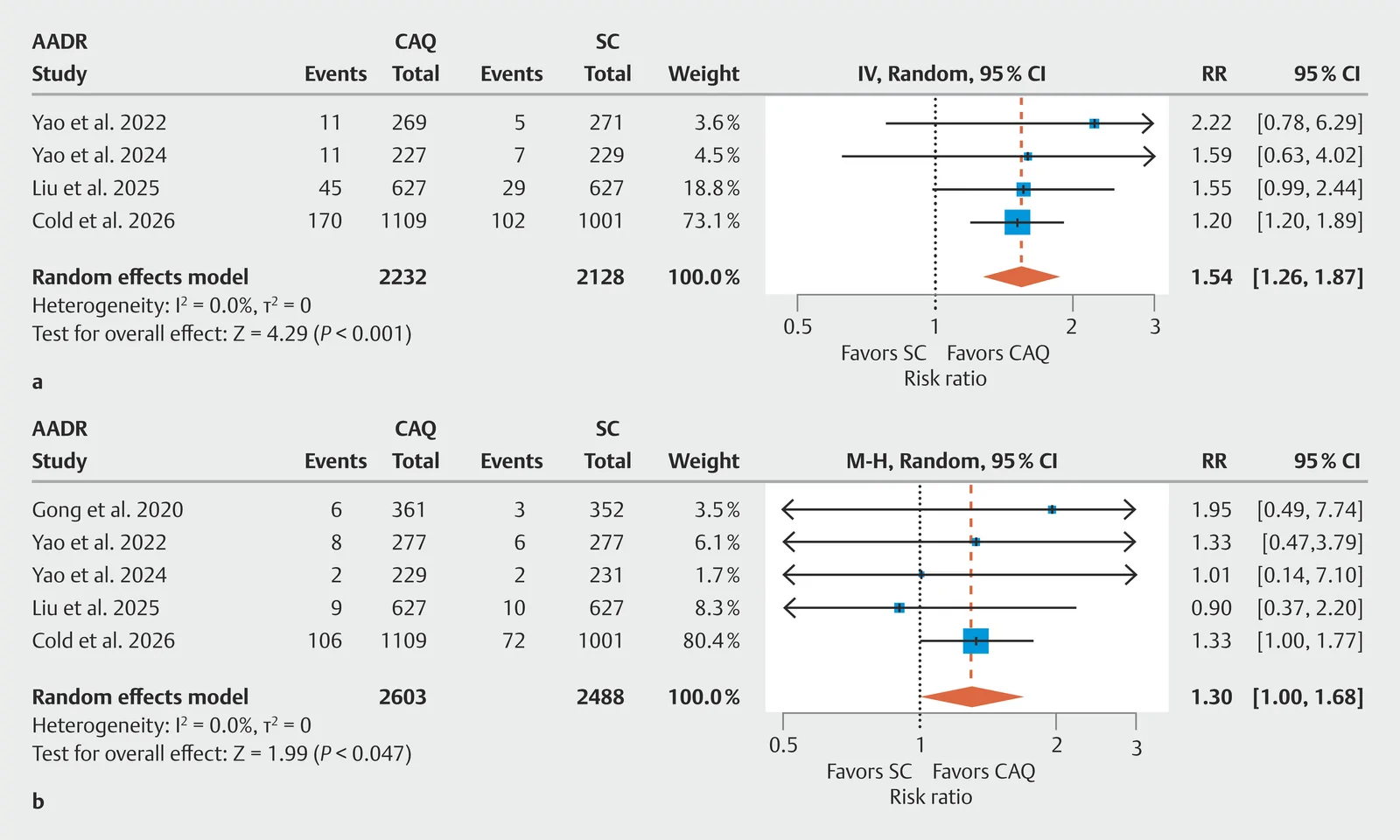
Forest plots for comparisons between computer-aided quality assurance (CAQ) intervention and standard colonoscopy. **a** Advanced adenoma detection rate (AADR). **b** Adenocarcinoma detection rate (ACDR) The overall effect indicated that the use of CAQ significantly increased AADR by 54% (risk ratio [RR] 1.54 [95%CI 1.26–1.87]; *P* < 0.001, *I ^2^* = 0.0%), and ACDR by 30% (RR 1.30 [95%CI 1.00–1.68]; *P* = 0.047, *I ^2^* = 0.0%). These results should be interpreted with caution as the non-detectable heterogeneity *I ^2^* = 0.0%) may reflect limited statistical power due to the small number of included studies rather than definitive clinical homogeneity. M-H, Mantel–Haenszel; SC, standard colonoscopy.

### Continuous secondary outcome measures (APC, PPC, and withdrawal time)

Compared with standard colonoscopy, CAQ increased APC by 0.14 adenomas (WMD 0.14 [95%CI 0.08–0.20]; *P* < 0.001, *I ^2^* = 53.2%) ( Table 3 , Fig. 4 ), and with omission of Liu et al. 2025 22 by 0.12 adenomas (WMD 0.12 [95%CI 0.08–0.16]; *P* < 0.001, *I ^2^* = 13.7%) ( Table 3 , **Fig. 9s** ). For PPC the increase was 0.31 polyps (WMD 0.31 [95%CI 0.17–0.44]; *P* < 0.001, *I ^2^* = 51.4%), and with omission of Cold et al. 23 0.34 polyps (WMD 0.34 [95%CI 0.24–0.45]; *P* < 0.001, *I ^2^* = 12.9%) ( Table 3 , **Fig. 10s** ). CAQ increased withdrawal time by 0.64 minutes (WMD 0.64 [95%CI 0.04–1.24]; *P* = 0.04, *I ^2^* = 96.1%) ( Fig. 4 ), and with omission of Liu et al. 2025 22 by 0.72 minutes (WMD 0.72 [95%CI 0.06–1.37]; *P* = 0.03, *I ^2^* = 91.2%) ( **Fig. 11s** ).

**Table:** **Table 3** Summary of meta-analysis outcomes comparing computer-aided quality assurance vs. standard colonoscopy. The table presents the pooled effect estimates (weighted mean differences) alongside their 95%CIs, calculated using a random-effects model. Due to the high statistical heterogeneity observed across the primary analyses, a leave-one-out sensitivity analysis was conducted, omitting the study that decreased the *I ^2^* value the most.

	Count, n/N ^1^ (weighted mean)		Weighted mean difference ^2^ [95%CI]	P value ^3^	I ^2^ , %	τ ^2^
	CAQ	Standard colonoscopy				
APC	1965/2895 (0.68)	1593/2792 (0.57)	0.14 [0.08–0.20]	<0.001	53.2	0.003
APC leave-one-out ^4^	1426/2268 (0.63)	1292/2165 (0.60)	0.12 [0.08–0.16]	<0.001	13.7	>0.001
PPC	3484/2895 (1.20)	2775/2792 (0.99)	0.31 [0.17–0.44]	<0.001	51.4	0.012
PPC leave-one-out ^5^	2378/1786 (1.33)	1669/1791 (0.93)	0.34 [0.24–0.45]	<0.001	12.9	0.002
Withdrawal time (minutes)	30 706/2895 (10.6)	27 130/2792 (9.72)	0.64 [0.04–1.24]	0.04	96.1	0.502
Withdrawal time leave-one-out (minutes) ^4^	26 455/2268 (11.7)	23 140/2165 (10.69)	0.72 [0.06–1.37]	0.03	91.2	0.490
Adenoma size						
≤5 mm	1010/2895 (0.35)	758/2792 (0.27)	0.07 [0.04–0.10]	<0.001	14.8	<0.001
≤5 mm leave-one-out ^6^	NA	NA	NA	NA	NA	NA
6–9 mm ^7,8^	336/2587 (0.13)	262/2477 (0.11)	0.01 [0.00–0.02]	0.005	0.0	0
≥10 mm ^8^	313/2587 (0.12)	176/2477 (0.07)	0.03 [0.02–0.05]	<0.001	45.1	>0.001
≥10 mm leave-one-out ^4,8^	303/2360 (0.13)	171/2248 (0.08)	0.02 [0.00–0.03]	0.049	50.7	<0.001
Subgroup differences ^6^				<0.001	65.6	<0.001
Adenoma location						
Cecum ^7^	133/2895 (0.05)	109/2792 (0.04)	0.00 [0.00–0.01]	0.30	0	0
Ascendens	373/2895 (0.13)	226/2792 (0.08)	0.03 [0.02–0.05]	<0.001	28.5	<0.001
Ascendens leave-one-out ^5^	141/1786 (0.08)	71/1791 (0.04)	0.03 [0.02–0.05]	<0.001	25.4	<0.001
Transverse	369/2895 (0.13)	248/2792 (0.09)	0.04 [0.02–0.05]	<0.001	41.5	<0.001
Transverse leave-one-out ^9,10^	344/2626 (0.13)	239/2521 (0.10)	0.03 [0.01–0.05]	0.003	43.3	<0.001
Descendens	178/2895 (0.06)	116/2792 (0.04)	0.02 [0.00–0.03]	0.015	31.6	<0.001
Descendens leave-one-out ^9,10^	170/2626 (0.06)	111/2521 (0.04)	0.01 [0.00–0.03]	0.046	31.7	<0.001
Sigmoid ^7^	481/2895 (0.17)	358/2792 (0.13)	0.03 [0.01–0.05]	<0001	0	0
Rectum ^7^	221/2895 (0.08)	190/2792 (0.07)	0.01 [0.00–0.02]	0.02	0	0
Subgroup differences ^6^				<0.001	47.9	<0.001
APC, adenomas per colonoscopy; CAQ, computer-aided quality assurance; PPC, polyps per colonoscopy. ^1^ n = count; N = total number of colonoscopies. ^2^ The random-effects model assign weight to the individual studies. The reported weighted mean difference is thereby derived by pooling individual study-level effects, not by subtracting aggregate group-level weighted means. ^3^ Random-effects model. ^4^ Omitting Liu et al. 2025 22 . ^5^ Omitting Cold et al. 2026 23 . ^6^ Leave-one-out sensitivity analysis not reported, as each omission resulted in * I ^2^* = 0.0%. ^7^ Leave-one-out analysis not applied as * I ^2^* = 0.0% for the original analysis. ^8^ Data not available from Su et al. 2020 21 or left out due to leave-one-out sensitivity analysis. ^9^ Omitting Yao et al. 2022 15 . ^10^ Leave-one-out sensitivity analysis resulted in an increased * I ^2^* , indicating omission of each study served as a homogenizing factor.						

**Fig. 4:**
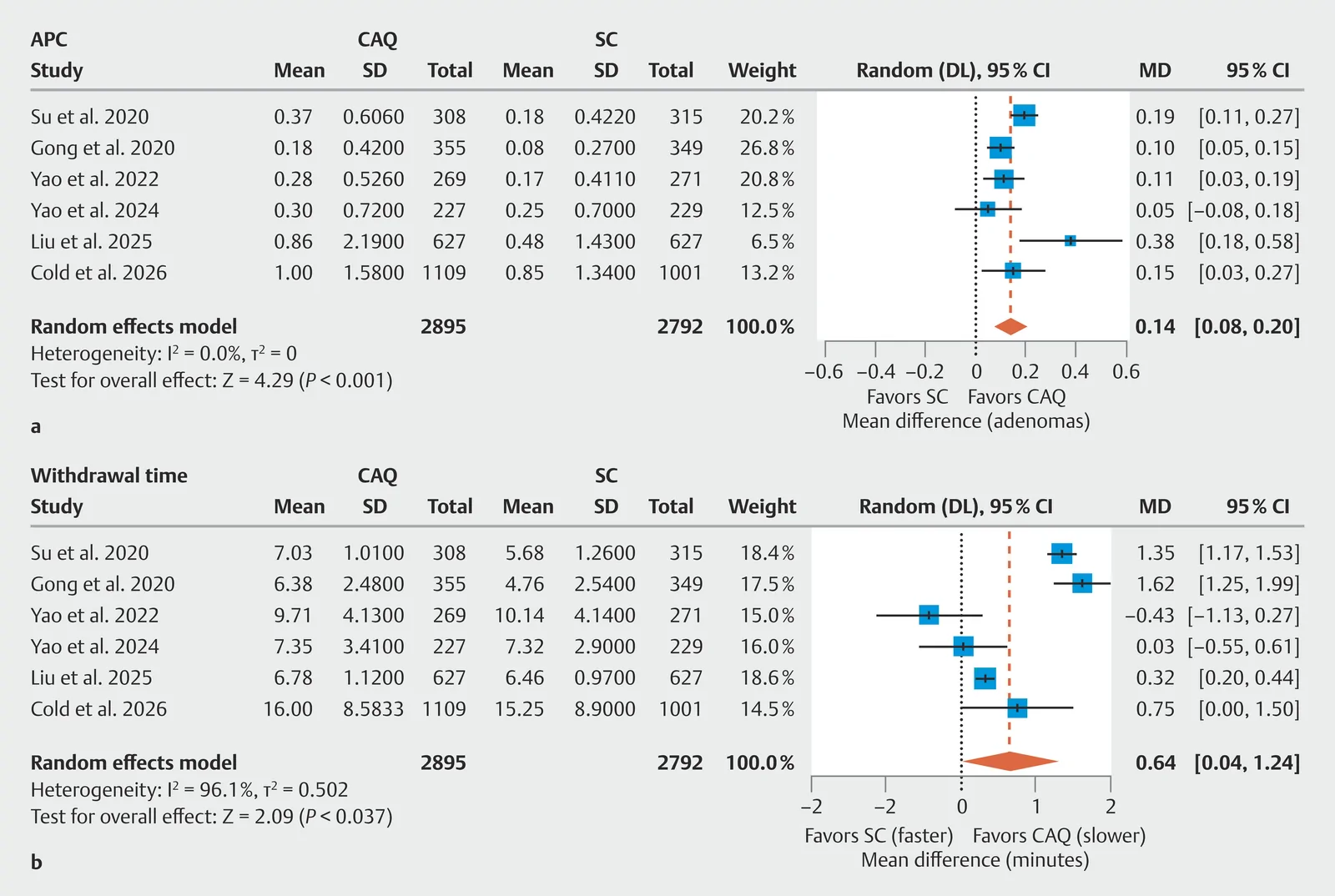
Forest plot for comparisons between computer-aided quality assurance (CAQ) intervention and standard colonoscopy. **a** Adenomas per colonoscopy (APC). **b** Withdrawal time. The overall effect indicated that the use of CAQ significantly increased APC by 0.14 adenomas per colonoscopy (mean difference [MD] 0.14 [95%CI 0.08–0.20]; *P* < 0.001, *I ^2^* = 53.2%), and withdrawal time by 0.64 minutes per colonoscopy (MD 0.64 [95%CI 0.04–1.24]; *P* = 0.04, *I ^2^* = 96.1%). Omission of Liu et al. 22 decreased the heterogeneity for APC (WMD 0.12 [95%CI 0.08–0.16]; *P* < 0.001, *I ^2^* = 13.7%), and led to a very subtle decrease in heterogeneity for withdrawal time (WMD 0.72 [95%CI 0.06–1.37]; *P* = 0.03, *I ^2^* = 91.2%) ( Table 3 ). DL, DerSimonian–Laird; MD, mean difference; SC, standard colonoscopy.

### Adenoma size

Compared with standard colonoscopy, CAQ increased detection of adenomas ≤5 mm by 0.07 adenomas per colonoscopy (WMD 0.07 [95%CI 0.04–0.10]; *P* < 0.001, *I ^2^* = 14.8%) ( Table 3 , **Fig. 12s** ). Omission of each study resulted in *I ^2^* = 0.0% ( **Fig. 13s** ). For adenomas 6–9 mm, the increase was 0.01 adenomas per colonoscopy (WMD 0.01 [95%CI 0.00–0.02]; *P* = 0.005, *I ^2^* = 0.0%). For adenomas ≥10 mm, the increase was 0.03 adenomas per colonoscopy (WMD 0.03 [95%CI 0.02–0.05]; *P* < 0.001, *I ^2^* = 45.1%); with omission of Liu et al. 22 the increase was 0.02 adenomas per colonoscopy (RR 0.02 [95%CI 0.00–0.03]; *P* = 0.049, *I ^2^* = 50.7%), indicating omission of each study served as a homogenizing factor. The effect of CAQ on adenoma detection varied significantly depending on lesion size (test for subgroup differences: *I ^2^* = 65.5%, *P* < 0.001) ( Table 3 ).

### Adenoma location

Compared with standard colonoscopy, CAQ did not increase adenoma detection in the cecum (WMD 0.00 [95%CI 0.00–0.01]; *P* = 0.30, *I ^2^* = 0.0%), but increased detection in the remaining locations ( Table 3 , **Fig. 14s** , **Fig. 15s** ). The effect of CAQ on adenoma location varied significantly depending on location (test for subgroup differences: *I ^2^* = 47.9%, *P* < 0.001).

### Analysis based on CAQ modality

The effect on ADR was consistent across all CAQ modalities, but several secondary outcomes were not statistically significant upon stratification due to the inherently reduced power of the smaller sample sizes ( **Table 3s** ).

## Discussion

Our analysis of 5687 colonoscopies across six RCTs showed an ADR improvement with CAQ of 43% (absolute increase +8.1%) compared with standard colonoscopy. The effect is numerically larger than that of CADe systems evaluated in a meta-analysis including 18 232 patients and 21 studies, where a 24% increase in ADR was found with CADe compared with standard colonoscopy 10 . However, extrapolating definitive superiority of CAQ over CADe systems from these indirect comparisons should be done cautiously. These estimates derive from different meta-analyses with varying baseline ADRs, populations, and study designs. The authors of the CADe meta-analysis concluded that the level of evidence was low, partly due to the high level of heterogeneity ( *I ^2^* = 70%) 10 . Omission of Cold et al. 23 increased the improvement of ADR slightly to 52% (absolute increase +8.9%) while the heterogeneity decreased substantially from *I ^2^* = 73.5% to *I ^2^* = 11.9%. The beneficial effect of CAQ compared with CADe is also apparent from the included four-group RCT by Yao et al. 15 , where CAQ was compared with CADe, but the authors were not able to detect a significant difference (ADR 24.5% vs. 21.3%; *P* = 0.52). However, we found that, compared with standard colonoscopy, CAQ increased AADR by 54% (+3.9%; *P* < 0.001, *I ^2^* = 0.0%), ACDR by 30% (+1.3%; *P* = 0.047, *I ^2^* = 0.0%), and adenomas ≥10 mm by 0.03 adenomas per colonoscopy ( *P* < 0.001, *I ^2^* = 45.1%), which are outcome measures that CADe does not improve 10 . Given that CADe can only evaluate what the endoscopists visualize, and not what the endoscopists fail to visualize, it has been proposed that CADe might only be beneficial for smaller adenomas 10 24 , while CAQ seemed to also be beneficial for larger adenomas. However, the results should be interpreted cautiously given the lower AADRs compared with ADRs and the different denominators across studies.

Yao et al. 15 demonstrated a combined effect of CADe+CAQ, which was greater than CADe alone (ADR 30.6% vs. 21.3%; *P* = 0.02), but not significantly greater than CAQ alone (30.6% vs. 24.5%; *P* = 0.21). As two included RCTs utilized combined CADe and CAQ systems, the individual effects of these systems cannot be distinguished, necessitating future evaluations of the respective modalities. Accordingly, the results of this meta-analysis should be interpreted cautiously considering two of the included RCTs utilized these integrated intervention designs, and the effect on the outcome measures cannot solely be attributed to the CAQ function alone.

The CAQ systems included in the current analysis aim to encourage a thorough inspection across the entire colon; CAQ increased APC by 0.14 adenomas ( *P* < 0.001, *I ^2^* = 53.2%), and APC increased for all the colon segments except the cecum ( Table 2 ). Alerting the endoscopist to excessive withdrawal speeds might not be beneficial in this initial phase of withdrawal, and only one of the included RCTs provided mapping of the mucosal inspection 22 . As CAQ prolonged withdrawal time by a mere 0.64 minutes compared with standard colonoscopy ( *P* = 0.04, *I ^2^* = 96.1%), the significant improvement in outcome measures is likely attributable to a more efficient inspection rather than simply an extended examination time. However, this proposed mechanism must be interpreted with caution. Five of the six included studies (ENDOANGEL and AQCS) included a withdrawal speedometer. This system directly influences withdrawal speed rather than explicitly mapping mucosal visualization. The heterogeneity for withdrawal time was high ( *I ^2^* = 96.1%) even with the omission of Liu et al. 22 (0.72 minutes; *P* = 0.03, *I ^2^* = 91.2%); this is important as the included RCTs defined withdrawal time differently ( Table 1 ). Another approach to prolonging the withdrawal time has been to inspect the ascending colon twice, which can improve the ADR 25 . CAQ improved adenomas in the ascending colon by 0.03 adenomas per colonoscopy ( *P* < 0.001, *I ^2^* = 28.5 %), but our analysis also showed subgroup differences between colon segments ( *P* < 0.001, *I ^2^* = 47.9%), indicating the benefit from CAQ might be greater for certain parts of the colon.

The recommendation for withdrawal time has recently been updated, increasing the minimum inspection time from 6 minutes to 8 minutes 5 . However, simply prolonging withdrawal time does not inherently guarantee a high-quality, steady procedure, as it may merely extend a mediocre inspection where certain parts are still missed 26 . In contrast, CAQ might ensure that the time spent translates into a more meticulous and systematic mucosal evaluation 27 28 ; however, other CAQ systems, such as fold examination quality, have not yet been evaluated in an RCT 13 14 . Consequently, future prospective trials are warranted to further evaluate the segment-specific performance and quality metrics of CAQ systems 29 .

Beyond conventional adenomas, CAQ also significantly improved overall polyp detection, which encompasses not only non-neoplastic lesions but also clinically important precursors such as serrated lesions. The increase in PDR was 24% ( *P* = 0.001, *I ^2^* = 70.5%), rising to 30% ( *P* < 0.001, *I ^2^* = 28.9%) following omission of Cold et al. 23 ( Table 2 , **Fig. 7s** ). Similarly, the increase in PPC was 0.31 polyps ( *P* < 0.001, *I ^2^* = 51.4%), and with the omission of Cold et al. 23 , 0.34 polyps ( *P* < 0.001, *I ^2^* = 12.9%). While PDR and PPC are valuable quality markers for mucosal inspection and serrated lesion detection 5 , increasing them does not exclusively guarantee a reduction in PCCRC to the same established degree as ADR.

No RCTs have examined how CAQ works in combination with computer-aided diagnosis (CADx). The resect-and-discard strategy using CADx for diminutive polyps (≤5 mm) offers no significant clinical benefit, as a meta-analysis found the proportion of polyps avoiding pathological assessment remained stagnant at 90% regardless of CADx assistance 30 . This lack of impact is attributed to the high baseline of high-confidence optical diagnoses (90%) already achieved by endoscopists, which leaves minimal room for technological improvement 30 . Furthermore, while CADx assistance slightly reduced the proportion of incorrectly predicted polyps, the marginal 1% difference is considered clinically negligible 30 . Improving the accuracy and explainability of CADx is essential to facilitating the safe implementation of the resect-and-discard strategy and with leaving non-neoplastic polyps in situ, thereby reducing unnecessary pathology costs and procedural interventions 31 .

### Strengths and limitations

Five of the six RCTs demonstrated a low risk of bias across all domains according to the RoB 2 tool ( Fig. 1 ). This finding is consistent with a systematic review and meta-analysis on AI-assisted colonoscopy, where 26 of 33 included trials (79%) exhibited a low risk of bias, and the four oldest RCTs in this study were rated identically 8 . There were “some concerns” about the study by Cold et al. 23 relating to the randomization process and deviations from intended interventions. This was due to its stepped-wedge cluster-randomized design, with only three clusters, and technical difficulties with the CAQ system, which prolonged the control period. Because blinding endoscopists to CAQ feedback is technologically impossible, all included trials carry an inherent risk of performance bias, such as the Hawthorne effect, which may not be fully reflected by standardized risk of bias assessment tools. Furthermore, while CAQ demonstrated significant efficacy in these RCTs, its true effectiveness in a daily clinical setting remains uncertain. Given that pragmatic CADe implementation studies have often failed to reproduce the ADR improvements observed in initial RCTs 32 33 , it is highly plausible that CAQ could face a similar attenuation of effect in routine clinical practice 24 . Although Cold et al. 23 was included as an unpublished preprint, its methodology and findings were acknowledged as robust through the statistical peer review before submission of this manuscript and accepted during the review process on June 1, 2026.

Previous meta-analyses have pooled CAQ with CADe RCTs, but with CAQ RCTs accounting for a minority of the studies included in the meta-analyses (3/11 and 4/33 studies, respectively) 8 34 , making the current study the first meta-analysis solely focusing on CAQ and including all currently available RCTs. Two of the included studies evaluated a combined CADe and CAQ system (AQCS), which could confound the isolated effect of CAQ; however, our secondary sensitivity analysis ( **Table 3s** ) evaluating each CAQ system individually confirmed the robustness of our primary grouped findings, indicating a class effect of CAQ on ADR. Furthermore, Cold et al. 23 used post-colonoscopy feedback rather than real-time feedback; to address this, the pooled effects grouped by specific system categories are detailed in **Table 3s** and through the leave-one-out analyses. While CADe primarily increased ADR through the detection of diminutive adenomas 10 , CAQ might have a better chance of reducing PCCRC by significantly improving AADR, ACDR, and adenomas ≥10 mm. A training intervention improving ADR has been linked to a reduction in PCCRC 35 . Follow-up analysis of PCCRC in the included RCTs should also be conducted.

Five of the six included RCTs were conducted in China, which may limit the generalizability of these findings to the rest of the world. The ADRs observed in these trials were relatively low compared with typical Western screening cohorts, which likely reflects regional differences in underlying population risk and baseline endoscopy quality. This geographical concentration is mirrored in the regulatory status of the evaluated technologies, with none holding US Food and Drug Administration or European Medicines Agency approval 36 . Additionally, there were notable variations in colonoscopy indications across the trials. While the Chinese studies included a heterogeneous mix of screening, surveillance, and symptomatic patients, only one trial was conducted exclusively in a Western screening population with positive fecal immunochemical tests, which reflected the higher ADRs in this RCT 23 . Consequently, while CAQ demonstrated a significant relative increase in ADR (+43%), the absolute increase (+8.1%) might differ when applied broadly to Western populations with a higher ADR. These differences in patient cohorts and indications further complicate the direct comparison and broad applicability of the pooled effect estimates to specific clinical settings. For instance, the relatively modest absolute improvements observed in the detection of only 0.03 adenomas ≥10 mm per colonoscopy ( *P* < 0.001, *I ^2^* = 45.1%) may reflect the rather low baseline detection rates (weighted mean CAQ vs. standard: 0.12 vs. 0.07 adenomas ≥10 mm) within these specific RCTs. The clinical value of CAQ might be more accurately appreciated when interpreted as a relative percentage increase, rather than a mere absolute gain (43% increase instead of 0.03 increase in adenomas per colonoscopy for adenomas ≥10 mm). Outcome reporting was assumed to be consistent for ADR, APC, etc. Although, three studies consistently defined AADR similarly, none provided explicit definitions for adverse events nor whether sessile serrated lesions were defined as only polyps, which is important given that they have precancerous characteristics. This variability highlights the critical need for future trials to adhere to standardized reporting frameworks 26 , to ensure robust comparability.

Several secondary analyses yielded an *I ^2^* = 0.0%. While this suggests that the observed variance across studies was consistent with sampling error rather than true clinical heterogeneity, these findings must be interpreted with caution. It is particularly relevant for outcomes with low event rates, such as ACDR and adverse events, where the high relative sampling error often masks potential between-study differences. The limited number of studies included in some sub-analyses thereby reduced the statistical power to detect between-study variance, meaning that low heterogeneity values may have reflected a lack of data to prove diversity rather than absolute clinical homogeneity. Additionally, publication bias of the included studies was not assessed, as more than 10 studies are needed to reliably assess funnel plots 37 .

## Conclusion

This first meta-analysis comparing CAQ with standard colonoscopy demonstrated a significant 43% increase in ADR. By encouraging more systematic mucosal visualization, CAQ targets what is not seen during colonoscopy, rather than merely recognizing already visualized lesions. The increase in ADR from meta-analyses (CAQ = 43% increase vs. CADe = 24% increase 10 ), might indicate that CAQ can provide a superior approach to quality improvement than CADe.

## Supplementary Material

Supplementary Material Zusatzmaterial
