# Supplementary material for: Computer-aided quality assurance versus standard colonoscopy: a systematic review and meta-analysis of randomized controlled trials
Source: Endoscopy. 2026 Aug 6;58(11):1275–86. doi: 10.1055/a-2904-8262 (PMC13759006; doi:10.1055/a-2904-8262)
Supplement: Supplementary file 1 [file 10-1055-a-2904-8262_29205403.pdf]

Thieme

Supplementary material

Computer-aided quality assurance versus standard colonoscopy: a systematic review and meta-analysis of randomized-controlled trials

Kristoffer Mazanti Cold, Martine S. Nielsen, Juana Maria Abdelreda, Flemming Bjerrum, Lars Konge.

Contents

Supplementary methods Search Strings ... 2
Table 1s 2020 PRISMA Checklist [1]. ... 3
Table 2s Description of the utilized CAQ feedback systems in the included RCTs. ... 6
Table 3s Outcomes grouped to CAQ intervention (ENDOANGEL [6-8], ACQS [9, 10], and CCC [11]). ... 7
Fig. 1s EMBASE: 596 hits (08th March 2026) ... 11
Fig. 2s CENTRAL: 584 when time limit 2020-present applied. ... 12
Fig. 3s: PRISMA chart [1], CAQ vs SC: SR and meta-analysis on RCTs ... 13
Forest Plots and Leave-one-out analyses ... 14
Fig. 4s ADR ... 14
Fig. 5s AADR ... 15
Fig. 6s ACDR ... 16
Fig. 7s PDR ... 17
Fig. 8s Adverse events ... 18
Fig. 9s APC ... 19
Fig. 10s PPC ... 20
Fig. 11s Withdrawal time ... 21
Fig. 12s Adenoma size ... 22
Fig. 13s Leave-one-out sensitivity analysis adenoma size. ... 23
Fig. 14s Adenoma location ... 24
Fig. 15s Leave-one-out sensitivity analyses for adenoma location ... 25

Endoscopy | DOI 10.1055/a-2904-8262 | © The Author(s).

Supplementary methods Search Strings

Searches were limited to 1st of January 2020 to present, as several systematic reviews on AI assisted colonoscopy RCTs have identified the first CAQ-feedback study was published in 2020 (Su et al. 2020 [2]) [3-5].

Pubmed: 191 hits (08th March 2026)

(colonoscopy or colonoscope or colonoscopist or sigmoidoscopy or "sigmoidoscopy"[Mesh] or colonoscopic* or "Colonoscopy"[Mesh]) and (competence or "Clinical Competence"[Mesh] or "Professional Competence"[Mesh] or assessment or "Process Assessment, Health Care"[Mesh] or "Outcome Assessment, Health Care"[Mesh] or validation or "Validation Studies as Topic"[Mesh] or tool or certification or "Certification"[Mesh] or "detection rate*" or "clinical skill*" or "Early detection of cancer" or "Early detection of cancer"[Mesh] or feedback) and (objective or automatic or unbiased or "artificial intelligence" or "artificial intelligence"[Mesh] or computerized or "AI based" or "AI assistance" or "deep learning" or "deep learning"[Mesh] or "Software Validation" or "Software Validation"[Mesh]) **Filters:** Randomized Controlled Trial, from 2020 - 2026

Endoscopy | DOI 10.1055/a-2904-8262 | © The Author(s).

Table 1s 2020 PRISMA Checklist [1].

Section and Topic	Item #	Checklist item	Location where item is reported (Page number in main document)
**TITLE**			
Title	1	Identify the report as a systematic review.	1
**ABSTRACT**			
Abstract	2	See the PRISMA 2020 for Abstracts checklist.	3-4
**INTRODUCTION**			
Rationale	3	Describe the rationale for the review in the context of existing knowledge.	5
Objectives	4	Provide an explicit statement of the objective(s) or question(s) the review addresses.	6
**METHODS**			
Eligibility criteria	5	Specify the inclusion and exclusion criteria for the review and how studies were grouped for the syntheses.	7-8
Information sources	6	Specify all databases, registers, websites, organisations, reference lists and other sources searched or consulted to identify studies. Specify the date when each source was last searched or consulted.	Supplemental material 2
Search strategy	7	Present the full search strategies for all databases, registers and websites, including any filters and limits used.	Supplemental material 2
Selection process	8	Specify the methods used to decide whether a study met the inclusion criteria of the review, including how many reviewers screened each record and each report retrieved, whether they worked independently, and if applicable, details of automation tools used in the process.	7-8
Data collection process	9	Specify the methods used to collect data from reports, including how many reviewers collected data from each report, whether they worked independently, any processes for obtaining or confirming data from study investigators, and if applicable, details of automation tools used in the process.	8-9
Data items	10a	List and define all outcomes for which data were sought. Specify whether all results that were compatible with each outcome domain in each study were sought (e.g. for all measures, time points, analyses), and if not, the methods used to decide which results to collect.	8-9
	10b	List and define all other variables for which data were sought (e.g. participant and intervention characteristics, funding sources). Describe any assumptions made about any missing or unclear information.	8-9
Study risk of bias assessment	11	Specify the methods used to assess risk of bias in the included studies, including details of the tool(s) used, how many reviewers assessed each study and whether they worked independently, and if applicable, details of automation tools used in the process.	9
Effect measures	12	Specify for each outcome the effect measure(s) (e.g. risk ratio, mean difference) used in the synthesis or presentation of results.	8-9
Synthesis methods	13a	Describe the processes used to decide which studies were eligible for each synthesis (e.g. tabulating the study intervention characteristics and comparing against the planned groups for each synthesis (item #5)).	8-9
	13b	Describe any methods required to prepare the data for presentation or synthesis, such as handling of missing summary statistics, or data conversions.	8-9

Endoscopy | DOI 10.1055/a-2904-8262 | © The Author(s).

Section and Topic	Item #	Checklist item	Location where item is reported (Page number in main document)
	13c	Describe any methods used to tabulate or visually display results of individual studies and syntheses.	8-9
	13d	Describe any methods used to synthesize results and provide a rationale for the choice(s). If meta-analysis was performed, describe the model(s), method(s) to identify the presence and extent of statistical heterogeneity, and software package(s) used.	8-9
	13e	Describe any methods used to explore possible causes of heterogeneity among study results (e.g. subgroup analysis, meta-regression).	8-9
	13f	Describe any sensitivity analyses conducted to assess robustness of the synthesized results.	8-9
Reporting bias assessment	14	Describe any methods used to assess risk of bias due to missing results in a synthesis (arising from reporting biases).	8-9
Certainty assessment	15	Describe any methods used to assess certainty (or confidence) in the body of evidence for an outcome.	8-9
**RESULTS**			
Study selection	16a	Describe the results of the search and selection process, from the number of records identified in the search to the number of studies included in the review, ideally using a flow diagram.	11
	16b	Cite studies that might appear to meet the inclusion criteria, but which were excluded, and explain why they were excluded.	NA
Study characteristics	17	Cite each included study and present its characteristics.	11
Risk of bias in studies	18	Present assessments of risk of bias for each included study.	12
Results of individual studies	19	For all outcomes, present, for each study: (a) summary statistics for each group (where appropriate) and (b) an effect estimate and its precision (e.g. confidence/credible interval), ideally using structured tables or plots.	12-13
Results of syntheses	20a	For each synthesis, briefly summarise the characteristics and risk of bias among contributing studies.	12-13
	20b	Present results of all statistical syntheses conducted. If meta-analysis was done, present for each the summary estimate and its precision (e.g. confidence/credible interval) and measures of statistical heterogeneity. If comparing groups, describe the direction of the effect.	12-13
	20c	Present results of all investigations of possible causes of heterogeneity among study results.	12-13
	20d	Present results of all sensitivity analyses conducted to assess the robustness of the synthesized results.	12-13
Reporting biases	21	Present assessments of risk of bias due to missing results (arising from reporting biases) for each synthesis assessed.	12-13
Certainty of evidence	22	Present assessments of certainty (or confidence) in the body of evidence for each outcome assessed.	12-13
**DISCUSSION**			
1	23a	Provide a general interpretation of the results in the context of other evidence.	14-18
	23b	Discuss any limitations of the evidence included in the review.	16-18

Endoscopy | DOI 10.1055/a-2904-8262 | © The Author(s).

Section and Topic	Item #	Checklist item	Location where item is reported (Page number in main document)
	23c	Discuss any limitations of the review processes used.	16-18
	23d	Discuss implications of the results for practice, policy, and future research.	14-18
**OTHER INFORMATION**			
Registration and protocol	24a	Provide registration information for the review, including register name and registration number, or state that the review was not registered.	8
	24b	Indicate where the review protocol can be accessed, or state that a protocol was not prepared.	8
	24c	Describe and explain any amendments to information provided at registration or in the protocol.	None
Support	25	Describe sources of financial or non-financial support for the review, and the role of the funders or sponsors in the review.	None
Competing interests	26	Declare any competing interests of review authors.	None
Availability of data, code and other materials	27	Report which of the following are publicly available and where they can be found: template data collection forms; data extracted from included studies; data used for all analyses; analytic code; any other materials used in the review.	Upon request

From: Page MJ, McKenzie JE, Bossuyt PM, Boutron I, Hoffmann TC, Mulrow CD, et al. The PRISMA 2020 statement: an updated guideline for reporting systematic reviews. BMJ 2021;372:n71. doi: 10.1136/bmj.n71

Table 2s Description of the utilized CAQ feedback systems in the included RCTs.

<table>
<tr><td>CAQ</td><td>Description</td></tr>
<tr><td>ENDOANGEL,
Gong et al. 2020 [6]
Yao et al. 2022 [7]
Yao et al. 2024 [8]</td><td>A live withdrawal speedometer that alarms endoscopists when withdrawing too quickly to ensure an even inspection of the mucosal wall throughout the withdrawal phase.

The alarm is based on pixel alteration and thereby the retraction speed of the scope.</td></tr>
<tr><td>AQCS (Automatic Quality Control System)
Su et al. 2020 [9]
Liu et al 2025 [10]</td><td>Consist of four aspects in the first study [9] and five in the later [10]:
1. Cecum identifier to automatically time the withdrawal phase
2. Withdrawal speedometer
3. AI bowel preparation assessment
4. CADe
5. Adequate colon inspection mapping (added for the later version, for Liu et al. 2025 [10]).</td></tr>
<tr><td>CCC (Copenhagen Colonoscopy Concept)
Cold et al. 2026 [11]</td><td>Consists of immediate feedback after each colonoscopy (OR feedback) and cumulative feedback available through a webpage (office feedback).

OR feedback This is automated feedback presented to the endoscopist immediately after each colonoscopy procedure. It consists of the Colonoscopy Retraction Score (CoRS), which is calculated using electromagnetic signals to map the scope's path. The score is composed of three sub-scores [3]: Retraction Distance: The cumulative distance the scope tip travels from the cecum to the anus. Tip Retraction: The total perpendicular deviation of the scope tip from a direct retraction line, serving as an indicator of fold inspection. Retraction Efficiency: An evaluation of the degree of scope rotation or twisting based on how closely individual coils follow the same path.

The CoRS score is based on ZYX-coordinates originating from the scope through magnetic endoscopic imaging. A previous study correlated CoRS to ADR [3].

Office feedback (Cumulative feedback): This is cumulative performance data that endoscopists can access throughout the study period through a dedicated web-page. It included cumulative CoRS scores in comparison to their peers in the RCT.</td></tr>
</table>

Endoscopy | DOI 10.1055/a-2904-8262 | © The Author(s).

Table 3s Outcomes grouped to CAQ intervention (ENDOANGEL [6-8], ACQS [9, 10], and CCC [11]).

	CAQ		SC					
Dichotomous outcomes.	Events (%)	Total Colonoscopies	Events (%)	Total Colonoscopies	RR [CI %] Random-effects model	P-value random effects model	I^2	τ^2
ADR Total	1004 (34.7%)	2895	741 (26.6%)	2792	1.43 [1.17-1.73]	P<0.001	73.5%	0.038
ADR ENDOANGEL	171 (20.1%)	851	113 (13.3%)	849	1.51 [1.13-2.01]	P=0.005	41.0%	0.026
ADR AQCS	294 (31.4%)	935	194 (20.6%)	942	1.53 [1.29-1.81]	P<0.001	11.7%	0.002
ADR CCC	539 (48.6%)	1109	434 (43.4%)	1001	1.12 [1.02-1.23]	P=0.016	NA	NA
[1,2]AADR Total	237 (10.6%)	2232	143 (6.7%)	2128	1.54 [1.26-1.87]	P<0.001	0%	0
[2]AADR ENDOANGEL	22 (4.4%)	496	12 (2.4%)	500	1.84 [0.92-3.68]	P=0.086	0.0%	0
[1]AADR AQCS	45 (7.2%)	627	29 (4.6%)	627	1.55 [0.99-2.44]	P=0.058	NA	NA
AADR CCC	170 (15.3%)	1109	102 (10.2%)	1001	1.50 [1.20-1.89]	P<0.001	NA	NA
[1,*]ACDR Total	131 (5.0%)	2603	93 (3.7%)	2488	1.30 [1.00-1.68]	P=0.047	0%	0
ACDR ENDOANGEL	16 (1.8%)	867	11 (1.3%)	860	1.44 [0.67-3.09]	P=0.354	0.0%	0
[1]ACDR AQCS	9 (1.4%)	627	10 (1.6%)	627	0.90 [0.37-2.20]	P=0.817	NA	NA
ACDR CCC	106 (9.6%)	1109	72 (7.2%)	1001	1.33 [1.00-1.77]	P=0.052	NA	NA
[5]PDR Total	1162 (51.2%)	2268	936 (43.2%)	2165	1.24 [1.09-1.42]	P=0.001	70.5%	0.015
PDR ENDOANGEL	443 (52.1%)	851	348 (41.0%)	849	1.26 [1.13-1.40]	P<0.001	10.8%	<0.001
[5]PDR AQCS	118 (38.3%)	308	80 (25.4%)	315	1.51 [1.19-1.91]	P<0.001	NA	NA
PDR CCC	601 (54.2%)	1109	508 (50.7%)	1001	1.07 [0.98-1.16]	P=0.115	NA	NA
[1,4]Adverse events Total	2 (0.1%)	2360	5 (0.2%)	2248	0.40 [0.07-2.23]	P=0.30	0	0
[4]Adverse events ENDOANGEL	0 (0.0%)	624	0 (0.0%)	620	NA	NA	NA	NA

Endoscopy | DOI 10.1055/a-2904-8262 | © The Author(s).

[1]Adverse events AQCS	1 (0.2%)	627	1 (0.2%)	627	1.00 [0.06-15.95]	P=1.000	NA	NA
Adverse events CCC	1 (0.1%)	1109	4 (0.4%)	1001	0.23 [0.03-2.02]	P=0.183	NA	NA
Continuous outcomes	Count, Weighted mean	Colonoscopies	Weighted mean	Colonoscopies	§Weighted mean difference [CI 95%]	P-value random effects model	I^2	τ^2
APC Total	1965 (0.68)	2895	1593 (0.57)	2792	0.14 [0.08-0.20]	P<0.001	53.2%	0.003
APC ENDOANGEL	207 (0.24)	851	130 (0.15)	849	0.10 [0.06-0.14]	P<0.001	0.0%	0
APC AQCS	652 (0.70)	935	357 (0.38)	942	0.26 [0.08-0.44]	P=0.005	65.3%	0.012
APC CCC	1106 (1.00)	1109	854 (0.85)	1001	0.15 [0.03-0.27]	P=0.018	NA	NA
PPC Total	3484 (1.20)	2895	2775 (0.99)	2792	0.31 [0.17-0.44]	P<0.001	51.4%	0.012
PPC ENDOANGEL	1329 (1.56)	851	971 (1.14)	849	0.44 [0.27-0.62]	P<0.001	0.0%	0
PPC AQCS	1049 (1.12)	935	698 (0.74)	942	0.29 [0.18-0.41]	P<0.001	19.8%	0.003
PPC CCC	1508 (1.36)	1109	1271 (1.27)	1001	0.09 [-0.09-0.27]	P=0.314	NA	NA
Withdrawal time (minutes) Total	30706 (10.6)	2895	27130 (9.74)	2792	0.64 [0.04-1.24]	P=0.037	96.1%	0.502
Withdrawal time ENDOANGEL	6545 (7.69)	851	6085 (7.17)	849	0.43 [-0.91-1.77]	P=0.529	94.6%	1.317
Withdrawal time AQCS	6416 (6.86)	935	5840 (6.20)	942	0.83 [-0.18-1.84]	P=0.106	98.9%	0.525
Withdrawal time CCC	17744 (16.00)	1109	15265 (15.25)	1001	0.75 [0.00-1.50]	P=0.049	NA	NA

The table corresponds to table 2 (summary of meta-analysis outcomes comparing CAQ vs. SC) but has included analysis of the subgroups according to the CAQ system utilized (ENDOANGEL, ACQS, and CCC). The table presents the pooled effect estimates— RR for dichotomous outcomes and weighted means for continuous outcomes—alongside their 95% Confidence Intervals (CI), calculated using a random-effects model. §The random-effects model assign weight to the individual studies. The reported weighted mean difference is thereby derived by pooling individual study-level effects, not by subtracting aggregate group-level weighted means. [1]Data not available from Su et al. 2020 [9]. [2]Gong et al. 2020 [6]. [3]Yao et al. 2022 [7]. [4]Yao et al. 2024 [8]. [5]Liu et al. 2025 [10]. [6]Cold et al. 2026 [11]. Abbreviations: AADR = Advanced Adenoma Detection Rate ACDR = Adenocarcinoma Detection Rate, ACQS = Automatic Quality Control System, ADR = Adenoma Detection Rate, APC = Adenomas Per Colonoscopy, CAQ = Computer-aided Quality Assurance, CCC = Copenhagen Colonoscopy Concept, CI = Confidence Interval, PDR = Polyp Detection Rate, PPC = Polyps Per Colonoscopy RR = Risk Ratio, SC = Standard Colonoscopy.

Comment on analysis based on CAQ-intervention systems:
While the overall pooled analysis demonstrated significant improvements across most primary and secondary outcomes, stratifying the results by the specific CAQ intervention (ENDOANGEL, AQCS, and CCC) inherently reduced the statistical power of the analysis due to dividing the total sampling size. Most notably the primary outcome measure of the

Endoscopy | DOI 10.1055/a-2904-8262 | © The Author(s).

analysis (ADR) was statistically significant across all CAQ systems. The same was true for APC. Consequently, the following outcome measures that were statistically significant in the overall pooled cohort lost their significance when evaluated within specific system categories. AADR: The significant overall increase in AADR appears largely driven by the CCC system (P<0.001). When evaluated independently, both ENDOANGEL (P=0.086) and AQCS (P=0.058) became statistically insignificant. ACDR: The overall improvement in ACDR was already modest (P=0.047), and this statistical significance was lost entirely across all three individual CAQ subgroups upon stratification. Withdrawal Time: The overall significant prolongation of withdrawal time (P=0.037) was primarily sustained by the CCC subgroup (P=0.049). In contrast, neither ENDOANGEL (P=0.53) nor AQCS (P=0.11) demonstrated a statistically significant increase in withdrawal time when analyzed independently. PDR and PPC: Conversely, while the overall findings for PDR and PPC were highly significant, these metrics became statistically insignificant specifically within the CCC subgroup (P=0.12 and P=0.31, respectively), despite robust significance in the ENDOANGEL and AQCS groups. Adverse events were not statistically significant in the overall analysis (P=0.30). Therefore, the lack of statistical significance for certain outcomes in these subgroups should be interpreted with caution. Rather than definitively indicating a lack of efficacy for a specific system, these shifts could be highly reflective of underpowered analyses resulting from grouping the available studies.

Endoscopy | DOI 10.1055/a-2904-8262 | © The Author(s).

References:
1. Page MJ, McKenzie JE, Bossuyt PM, Boutron I, Hoffmann TC, Mulrow CD, et al. The PRISMA 2020 statement: an updated guideline for reporting systematic reviews. Bmj. 2021;372:n71.
2. Su JR, Li Z, Shao XJ, Ji CR, Ji R, Zhou RC, et al. Impact of a real-time automatic quality control system on colorectal polyp and adenoma detection: a prospective randomized controlled study (with videos). Gastrointest Endosc. 2020;91(2):415-24.e4.
3. Cold KM, Vamadevan A, Vilmann AS, Svendsen MBS, Konge L, Bjerrum F. Computer-aided quality assessment of endoscopist competence during colonoscopy: A systematic review. Gastrointestinal Endoscopy. 2024.
4. Hassan C, Spadaccini M, Iannone A, Maselli R, Jovani M, Chandrasekar VT, et al. Performance of artificial intelligence in colonoscopy for adenoma and polyp detection: a systematic review and meta-analysis. Gastrointest Endosc. 2021;93(1):77-85.e6.
5. Lou S, Du F, Song W, Xia Y, Yue X, Yang D, et al. Artificial intelligence for colorectal neoplasia detection during colonoscopy: a systematic review and meta-analysis of randomized clinical trials. EClinicalMedicine. 2023;66:102341.
6. Gong D, Wu L, Zhang J, Mu G, Shen L, Liu J, et al. Detection of colorectal adenomas with a real-time computer-aided system (ENDOANGEL): a randomised controlled study. The lancet Gastroenterology & hepatology. 2020;5(4):352-61.
7. Yao L, Zhang L, Liu J, Zhou W, He C, Zhang J, et al. Effect of an artificial intelligence-based quality improvement system on efficacy of a computer-aided detection system in colonoscopy: a four-group parallel study. Endoscopy. 2022;54(8):757-68.
8. Yao L, Li X, Wu Z, Wang J, Luo C, Chen B, et al. Effect of artificial intelligence on novice-performed colonoscopy: a multicenter randomized controlled tandem study. Gastrointest Endosc. 2024; 99: 91–99.e9.
9. Su J-R, Li Z, Shao X-J, Ji C-R, Ji R, Zhou R-C, et al. Impact of a real-time automatic quality control system on colorectal polyp and adenoma detection: a prospective randomized controlled study (with videos). Gastrointestinal endoscopy. 2020;91(2):415-24. e4.
10. Liu J, Zhou R, Liu C, Liu H, Cui Z, Guo Z, et al. Automatic Quality Control System and Adenoma Detection Rates During Routine Colonoscopy: A Randomized Clinical Trial. JAMA Netw Open. 2025;8(1):e2457241.
11. Cold KM, Vamadevan A, Heen A, Vilmann AS, Rasmussen M, Svendsen MB, et al. A Computer Aided Quality Feedback Bundle Improves Colonoscopists' Adenoma Detection Rate: A Multicentre Cluster-Randomized Controlled Trial. 2026. Available at SSRN 5172465 Second round of review submitted 18 Mar 2026. 2025.

Endoscopy | DOI 10.1055/a-2904-8262 | © The Author(s).

Fig. 1s EMBASE: 596 hits (08th March 2026)

1.	exp colonoscopy/
2.	(colonoscopy or colonoscope or colonoscopist or colonoscopic*).mp. [mp=title, abstract, heading word, drug trade name, original title, device manufacturer, drug manufacturer, device trade name, keyword heading word, floating subheading word, candidate term word]
3.	1 or 2
4.	exp clinical competence/
5.	professional competence/
6.	health care quality/
7.	outcome assessment/
8.	exp validation study/
9.	certification/
10.	early cancer diagnosis/
11.	(competence or assessment or validation or tool or certification or detection rate* or clinical skill* or Early detection cancer or feedback).mp. [mp=title, abstract, heading word, drug trade name, original title, device manufacturer, drug manufacturer, device trade name, keyword heading word, floating subheading word, candidate term word]
12.	4 or 5 or 6 or 7 or 8 or 9 or 10 or 11
13.	exp artificial intelligence/
14.	deep learning/
15.	software validation/
16.	(objective or automatic or unbiased or artificial intelligence or computerized or AI based or AI assistance or deep learning or Software Validation).mp. [mp=title, abstract, heading word, drug trade name, original title, device manufacturer, drug manufacturer, device trade name, keyword heading word, floating subheading word, candidate term word]
17.	13 or 14 or 15 or 16
18.	3 and 12 and 17
19.	limit 18 to conference abstracts
20.	18 not 19
21.	limit 20 to english language
22.	randomized controlled trial/
23.	randomization/
24.	double blind procedure/
25.	single blind procedure/
26.	crossover procedure/
27.	(random* or factorial or crossover or cross-over or placebo or assign* or allocat* or volunteer*).ti,ab.
28.	22 or 23 or 24 or 25 or 26 or 27
29.	21 and 28
30.	limit 29 to yr="2020 -Current"

Fig. 2s CENTRAL: 584 when time limit 2020-present applied.

#1	[mh Colonoscopy]	3094
#2	(colonoscopy OR colonoscope OR colonoscopist OR colonoscopic* OR sigmoidoscopy):ti,ab,kw	10839
#3	#1 OR #2	10839
#4	[mh "Clinical Competence"]	5630
#5	[mh "Professional Competence"]	5948
#6	[mh "Process Assessment, Health Care"]	399
#7	[mh "Outcome Assessment, Health Care"]	222099
#8	[mh Certification]	89
#9	[mh "Early Detection of Cancer"]	2806
#10	competence:ti,ab,kw OR assessment:ti,ab,kw OR validation:ti,ab,kw OR tool:ti,ab,kw OR certification:ti,ab,kw OR feedback:ti,ab,kw OR performance:ti,ab,kw OR training:ti,ab,kw OR skill:ti,ab,kw OR "detection rate":ti,ab,kw OR "clinical skill":ti,ab,kw OR "early detection of cancer":ti,ab,kw	612862
#11	#4 OR #5 OR #6 OR #7 OR #8 OR #9 OR #10	759205
#12	[mh "Artificial Intelligence"]	3898
#13	[mh "Deep Learning"]	409
#14	objective:ti,ab,kw OR automatic:ti,ab,kw OR unbiased:ti,ab,kw OR "artificial intelligence":ti,ab,kw OR AI:ti,ab,kw OR "AI-assisted":ti,ab,kw OR "AI based":ti,ab,kw OR computerized:ti,ab,kw OR "machine learning":ti,ab,kw OR "deep learning":ti,ab,kw OR algorithm:ti,ab,kw OR automated:ti,ab,kw OR CAQ:ti,ab,kw OR "computer aided quality assurance":ti,ab,kw OR "computer-aided quality assurance":ti,ab,kw OR "software validation":ti,ab,kw	428772
#15	#12 OR #13 OR #14	429815
#16	#3 AND #11 AND #15	1172

Endoscopy | DOI 10.1055/a-2904-8262 | © The Author(s).

Fig. 3s: PRISMA chart [1], CAQ vs SC: SR and meta-analysis on RCTs

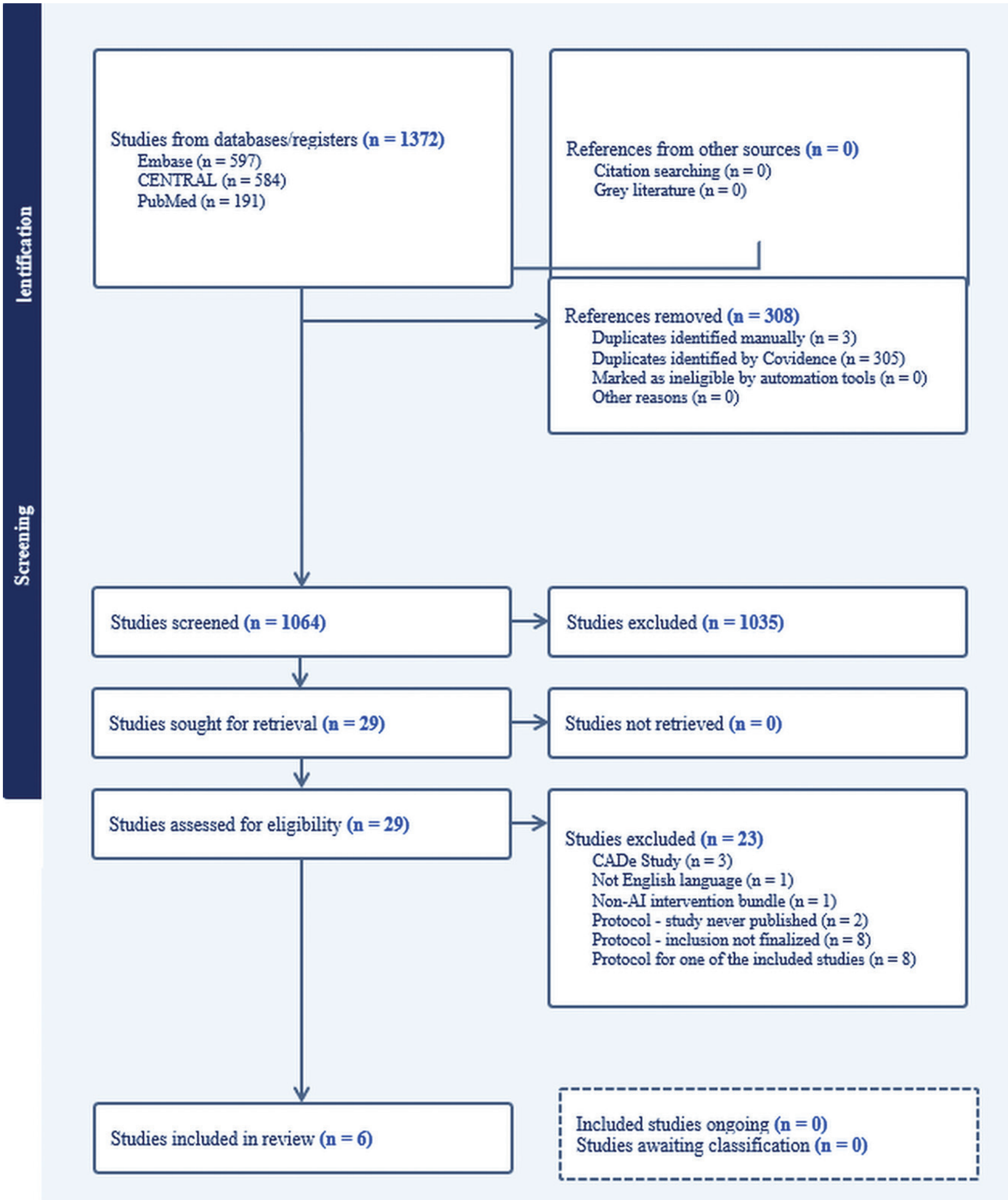

Endoscopy | DOI 10.1055/a-2904-8262 | © The Author(s).

Forest Plots and Leave-one-out analyses

Dichotomous Outcome Measures:
Fig. 4s ADR

(a) Forest plot comparing ADR between CAQ intervention and SC. The overall effect indicated that the use of CAQ significantly increased ADR by 43% compared to SC (RR 1.43; 95% CI [1.17; 1.73], P<0.001). Given the substantial heterogeneity observed in this primary analysis (I^2 = 73.5%), a leave-one-out sensitivity analysis was conducted to identify sources of variance from individual studies. **(b)** Leave-one-out sensitivity analysis table. Each row demonstrated the recalculated metrics when the specified study was omitted from the meta-analysis. Noticeably, omitting Cold et al. reduced the heterogeneity remarkably (from I^2=73.5% to I^2=11.9%). Omission of a single study kept resulting in a significant RR (range RR: 1.35-1.52) favoring CAQ to SC.

Abbreviations: ADR = Adenoma Detection Rate; CAQ = Computer-aided Quality Assurance; CI = Confidence Interval; M-H = Mantel-Haenszel; RR = Risk Ratio; SC = Standard Colonoscopy.

New figs

a, Forest Plot

	CAQ		SC					
ADR	Events	Total	Events	Total	Weight	M-H, Random, 95% CI	RR	95% CI
Su et al. 2020	89	308	52	315	15.8%		1.75	[1.29; 2.37]
Gong et al. 2020	58	355	27	349	11.3%		2.11	[1.37; 3.25]
Yao et al. 2022	66	269	49	271	14.8%		1.36	[0.98; 1.89]
Yao et al. 2024	47	227	37	229	12.7%		1.28	[0.87; 1.89]
Liu et al. 2025	205	627	142	627	21.0%		1.44	[1.20; 1.73]
Cold et al. 2026	539	1109	434	1001	24.4%		1.12	[1.02; 1.23]
**Random effects model**		**2895**		**2792**	**100.0%**		**1.43**	**[1.17; 1.73]**

Heterogeneity: I^2 = 73.7%, τ^2 = 0.038
Test for overall effect: z = 3.58 (p < 0.001)

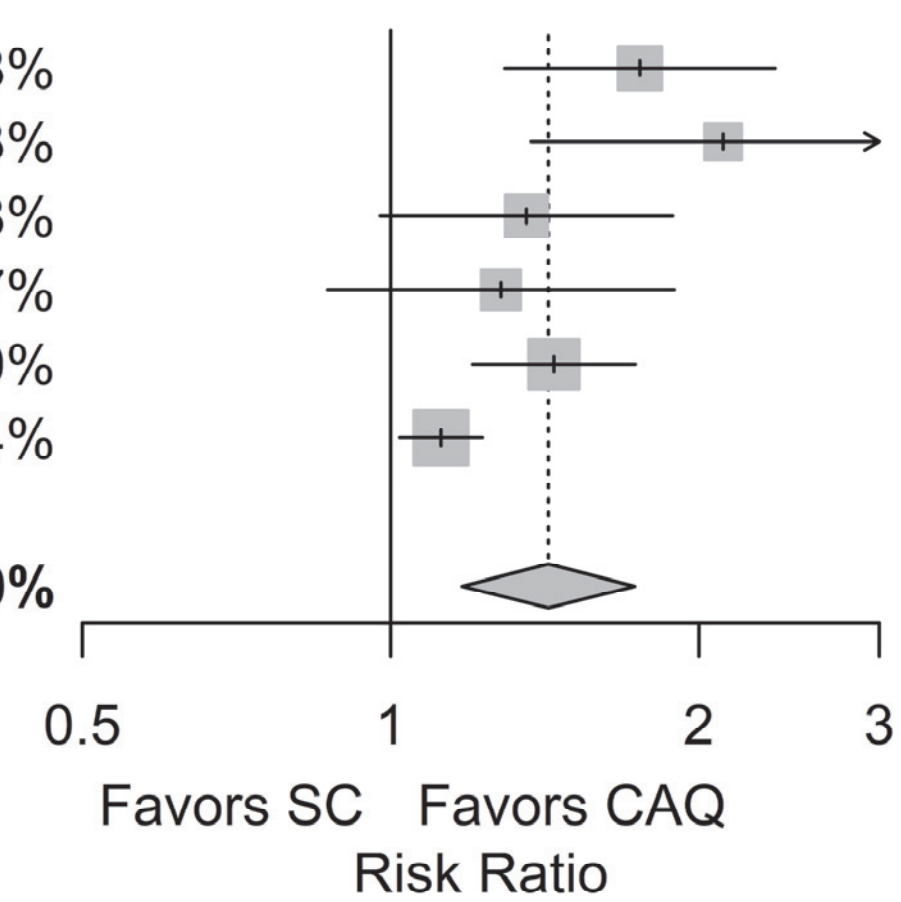

b

b, ADR Leave-one-out sensitivity analysis

Study omitted	RR	95% CI	P-value	I^2	Tau^2
Omitting Su et al., 2020	1.36	[1.12; 1.65]	0.002	69.4%	0.029
Omitting Gong et al., 2020	1.35	[1.13; 1.61]	<0.001	67.5%	0.025
Omitting Yao et al., 2022	1.44	[1.16; 1.80]	0.001	77.7%	0.044
Omitting Yao et al., 2024	1.45	[1.17; 1.81]	<0.001	78.0%	0.043
Omitting Liu et al., 2025	1.43	[1.12; 1.83]	0.004	73.7%	0.05
Omitting Cold et al., 2026	1.52	[1.32; 1.75]	<0.001	11.9%	0.003

Endoscopy | DOI 10.1055/a-2904-8262 | © The Author(s).

Fig. 5s AADR

Forest plot comparing AADR between CAQ intervention and SC. The overall effect indicated that the use of CAQ significantly increased AADR by 54% (1.54 [1.26; 1.87], P<0.001, I2= 0.0%). These results should be interpreted with caution as the non-detectable heterogeneity I^2=0.0%) may reflect limited statistical power due to the small number of included studies rather than definitive clinical homogeneity.

Abbreviations: AADR = Advanced Adenoma Detection Rate; CAQ = Computer-aided Quality Assurance; CI = Confidence Interval; M-H = Mantel-Haenszel; RR = Risk Ratio; SC = Standard Colonoscopy.

**AADR**		**CAQ**		**SC**				
**Study**	**Events**	**Total**	**Events**	**Total**	**Weight**	**M-H, Random, 95% CI**	**RR**	**95% CI**
Yao et al. 2022	11	269	5	271	3.6%		2.22	[0.78; 6.29]
Yao et al. 2024	11	227	7	229	4.5%		1.59	[0.63; 4.02]
Liu et al. 2025	45	627	29	627	18.8%		1.55	[0.99; 2.44]
Cold et al. 2026	170	1109	102	1001	73.1%		1.50	[1.20; 1.89]
**Random effects model**		**2232**		**2128**	**100.0%**		**1.54**	**[1.26; 1.87]**

Heterogeneity: $I^2 = 0.0\%$, $\tau^2 = 0$
Test for overall effect: $z = 4.29$ ($p < 0.001$)

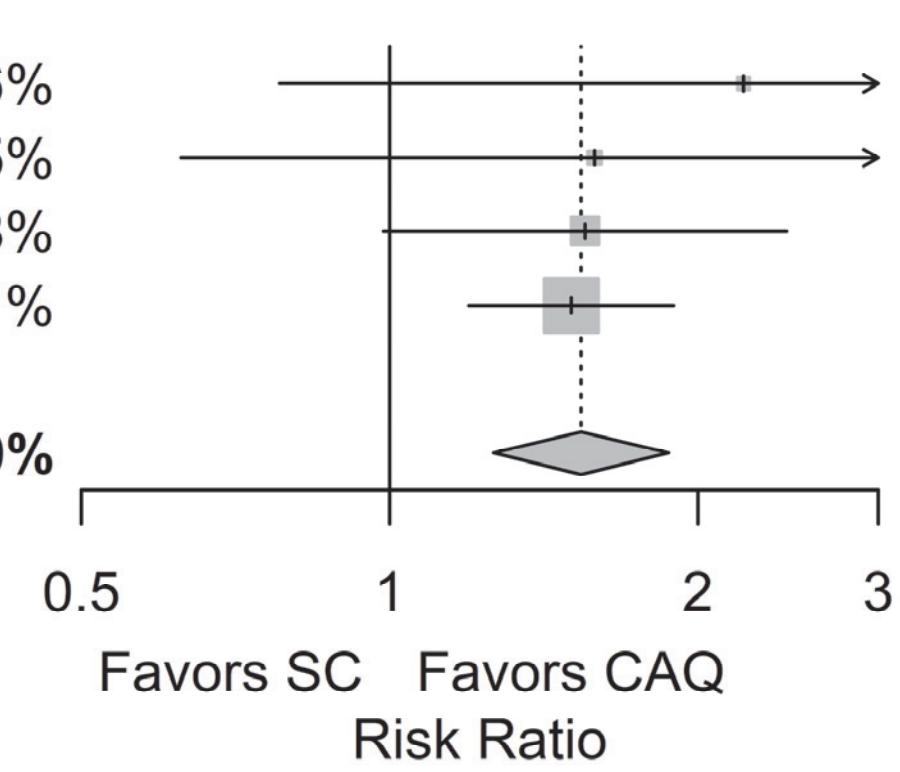

Endoscopy | DOI 10.1055/a-2904-8262 | © The Author(s).

Fig. 6s ACDR

Forest plot comparing ACDR between CAQ intervention and SC. The overall effect indicated that the use of CAQ significantly increased ACDR by 30% (1.30 [1.00; 1.68], P=0.047, I^2= 0.0%). These results should be interpreted with caution as the non-detectable heterogeneity I^2=0.0%) may reflect limited statistical power due to the small number of included studies rather than definitive clinical homogeneity.

Abbreviations: ACDR = Adenocarcinoma Detection Rate; CAQ = Computer-aided Quality Assurance; CI = Confidence Interval; M-H = Mantel-Haenszel; RR = Risk Ratio; SC = Standard Colonoscopy.

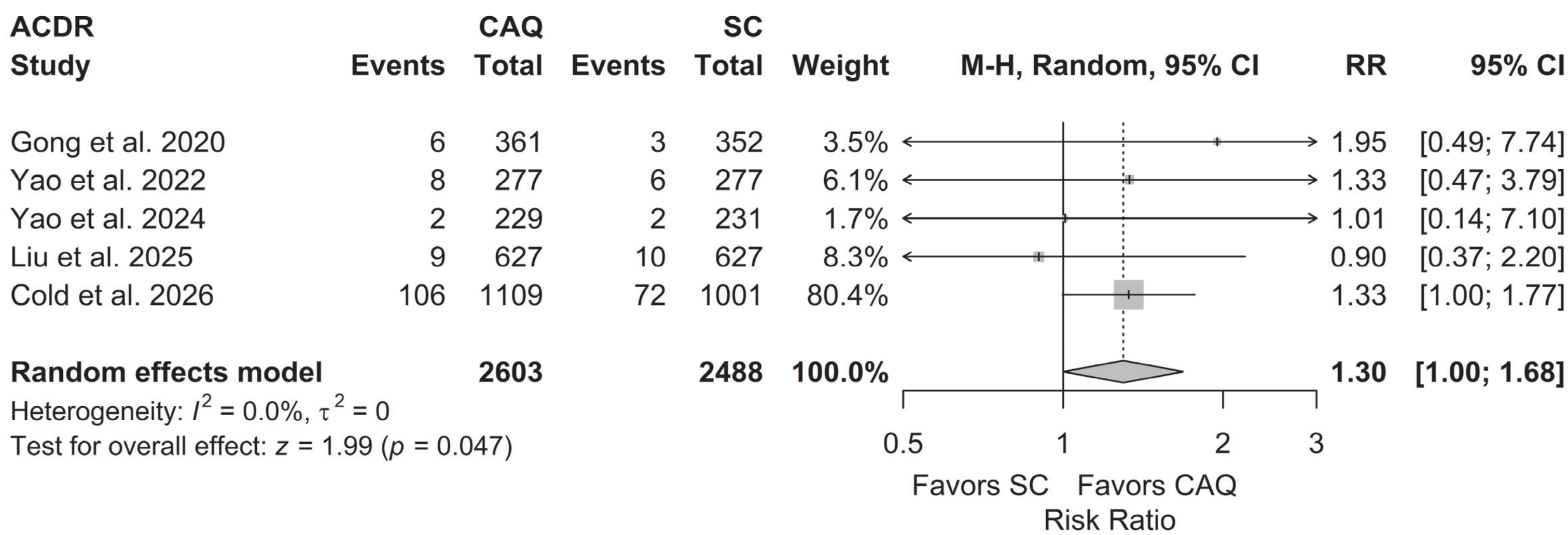

Endoscopy | DOI 10.1055/a-2904-8262 | © The Author(s).

Fig. 7s PDR

(a) Forest plot comparing PDR between CAQ intervention and SC. The overall effect indicated that the use of CAQ significantly increased PDR by 24% compared to SC (RR 1.24; 95% CI [1.09; 1.42], P=0.001). Given the substantial heterogeneity observed in this primary analysis (I^2 = 70.5%), a leave-one-out sensitivity analysis was conducted to identify sources of variance from individual studies. **(b)** Leave-one-out sensitivity analysis table. Each row demonstrated the recalculated metrics when the specified study was omitted from the meta-analysis. Noticeably, omitting Cold et al. reduced the heterogeneity remarkably (from I^2=70.5% to I^2=28.9%). Omission of a single study kept resulting in a significant RR (range RR: 1.05-1.51) favoring CAQ to SC.

Abbreviations: PDR = Polyp Detection Rate; CAQ = Computer-aided Quality Assurance; CI = Confidence Interval; M-H = Mantel-Haenszel; RR = Risk Ratio; SC = Standard Colonoscopy.

a

PDR	CAQ		SC					
Study	Events	Total	Events	Total	Weight	M-H, Random, 95% CI	RR	95% CI
Su et al. 2020	118	308	80	315	15.2%		1.51	[1.19; 1.91]
Gong et al. 2020	166	355	118	349	18.8%		1.38	[1.15; 1.66]
Yao et al. 2022	144	269	113	271	19.2%		1.28	[1.07; 1.54]
Yao et al. 2024	133	227	117	229	20.1%		1.15	[0.97; 1.36]
Cold et al. 2026	601	1109	508	1001	26.7%		1.07	[0.98; 1.16]
**Random effects model**		**2268**		**2165**	**100.0%**		**1.24**	**[1.09; 1.42]**

Heterogeneity: I^2 = 70.7%, τ^2 = 0.015

Test for overall effect: z = 3.22 (p = 0.001)

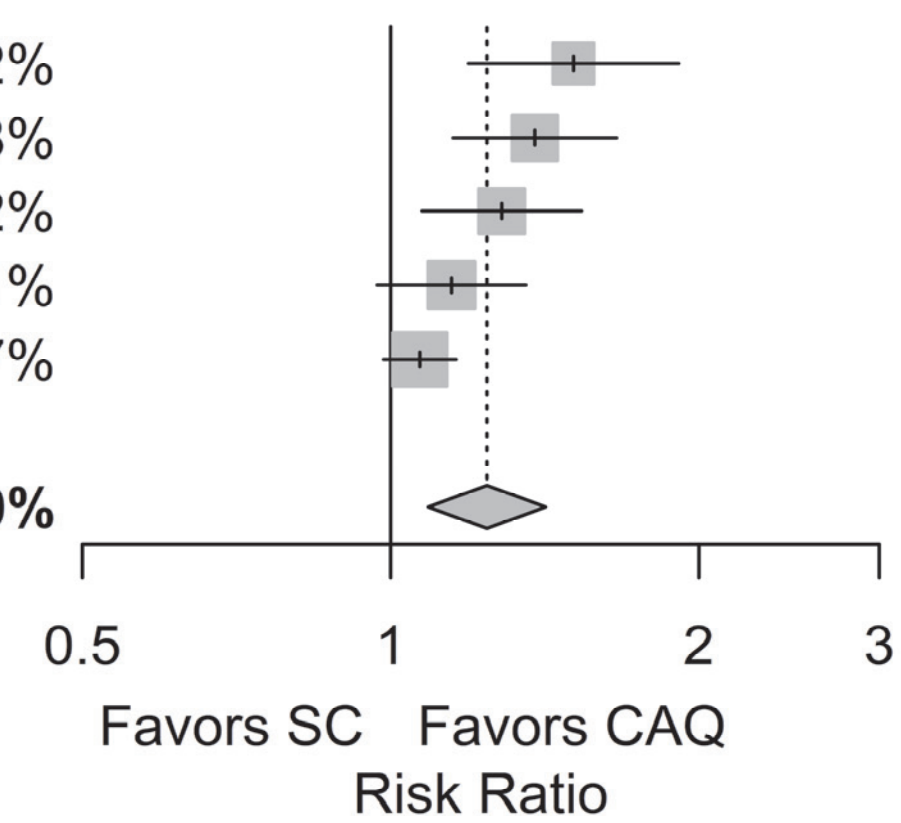

b

PDR Leave-one-out sensitivity analysis

Study omitted	RR	95% CI	P-value	I^2	Tau^2
Omitting Su et al., 2020	1.19	[1.06; 1.35]	0.005	64.2%	0.010
Omitting Gong et al., 2020	1.21	[1.05; 1.39]	0.008	68.6%	0.013
Omitting Yao et al., 2022	1.24	[1.06; 1.45]	0.009	75.4%	0.019
Omitting Yao et al., 2024	1.28	[1.08; 1.51]	0.005	77.8%	0.023
Omitting Cold et al., 2026	1.30	[1.16; 1.46]	<0.001	28.9%	0.004

Endoscopy | DOI 10.1055/a-2904-8262 | © The Author(s).

Fig. 8s Adverse events

Forest plot comparing adverse events between CAQ intervention and SC. The overall effect indicated that the use of CAQ did not impact adverse events significantly (RR [CI 95%]: 0.40 [0.07; 2.23], P=0.047, I^2= 0.0%). These results should be interpreted with caution as the non-detectable heterogeneity I^2=0.0%) may reflect limited statistical power due to the small number of included studies rather than definitive clinical homogeneity.

Abbreviations: CAQ = Computer-aided Quality Assurance; CI = Confidence Interval; M-H = Mantel-Haenszel; RR = Risk Ratio; SC = Standard Colonoscopy.

Adverse Events	CAQ		SC					
**Study**	**Events**	**Total**	**Events**	**Total**	**Weight**	**M-H, Random, 95% CI**	**RR**	**95% CI**
Gong et al. 2020	0	355	0	349	0.0%			
Yao et al. 2022	0	269	0	271	0.0%			
Liu et al. 2025	1	627	1	627	38.5%		1.00	[0.06; 15.95]
Cold et al. 2026	1	1109	4	1001	61.5%		0.23	[0.03; 2.02]
**Random effects model**		**2360**		**2248**	**100.0%**		**0.40**	**[0.07; 2.23]**

Heterogeneity: $I^2 = 0.0\%$, $\tau^2 = 0$

Test for overall effect: $z = -1.05$ ($p = 0.296$)

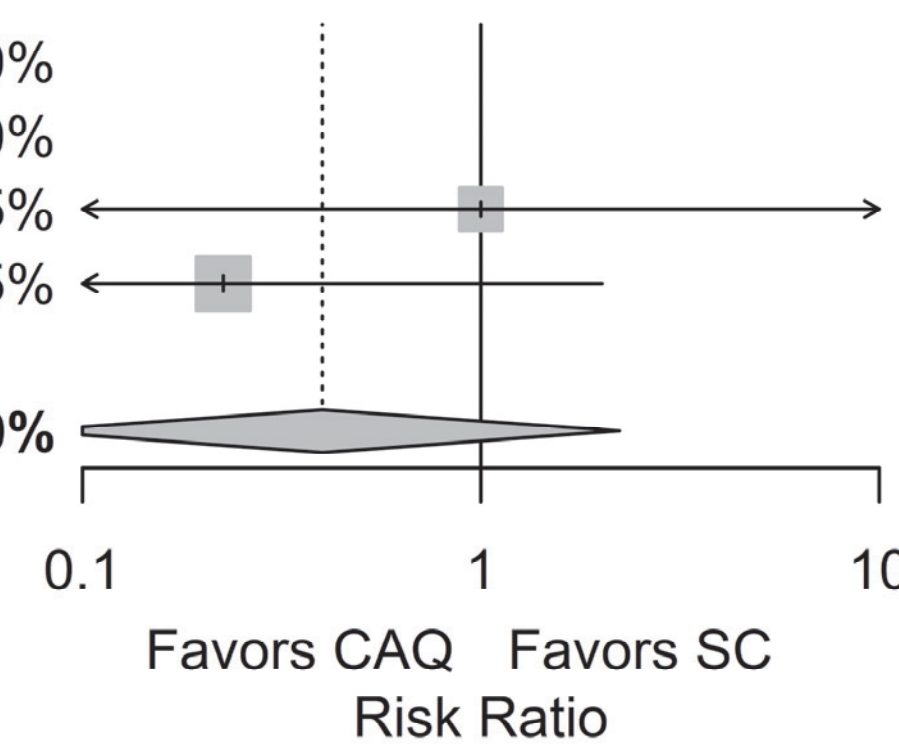

Endoscopy | DOI 10.1055/a-2904-8262 | © The Author(s).

Continuous outcome measures

Fig. 9s APC

(a) Forest plot comparing APC between CAQ intervention and SC. The overall effect indicated that the use of CAQ significantly increased APC by 0.14 adenomas to SC (WMD: 0.14; 95% CI [0.08; 0.02], P<0.001). Given the substantial heterogeneity observed in this primary analysis (I^2 = 53.2%), a leave-one-out sensitivity analysis was conducted to identify sources of variance from individual studies. **(b)** Leave-one-out sensitivity analysis table. Each row demonstrated the recalculated metrics when the specified study was omitted from the meta-analysis. Noticeably, omitting Liu et al. reduced the heterogeneity remarkably (from I^2=53.2% to I^2=13.7%). Omission of a single study kept resulting in a significant WMD (range WMD: 0.06-0.23) favoring CAQ to SC.

Abbreviations: APC = Adenoma Per Colonoscopy; CAQ = Computer-aided Quality Assurance; CI = Confidence Interval; DL = DerSimonian-Laird; SC = Standard Colonoscopy, WMD = Weighted Mean Difference.

a

APC	CAQ			SC						
**Study**	**Mean**	**SD**	**Total**	**Mean**	**SD**	**Total**	**Weight**	**Random (DL), 95% CI**	**MD**	**95% CI**
Su et al. 2020	0.37	0.6060	308	0.18	0.4220	315	20.2%		0.19	[0.11; 0.27]
Gong et al. 2020	0.18	0.4200	355	0.08	0.2700	349	26.8%		0.10	[0.05; 0.15]
Yao et al. 2022	0.28	0.5260	269	0.17	0.4110	271	20.8%		0.11	[0.03; 0.19]
Yao et al. 2024	0.30	0.7200	227	0.25	0.7000	229	12.5%		0.05	[-0.08; 0.18]
Liu et al. 2025	0.86	2.1900	627	0.48	1.4300	627	6.5%		0.38	[0.18; 0.58]
Cold et al. 2026	1.00	1.5800	1109	0.85	1.3400	1001	13.2%		0.15	[0.03; 0.27]
**Random effects model**			**2895**			**2792**	**100.0%**		**0.14**	**[0.08; 0.20]**

Heterogeneity: I^2 = 53.5%, τ^2 = 0.003

Test for overall effect: z = 4.73 (p < 0.001)

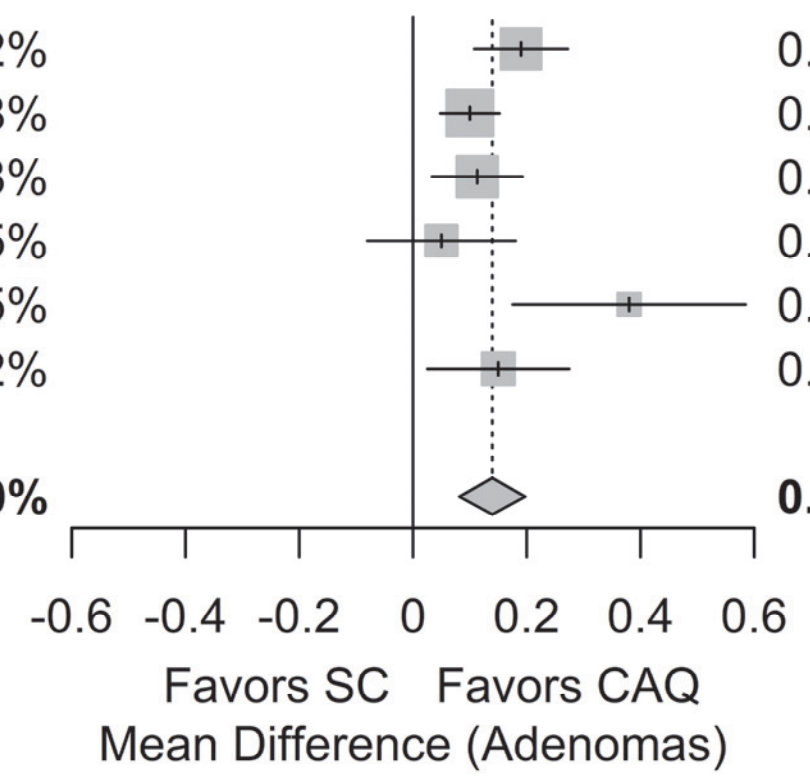

b

APC Leave-one-out sensitivity analysis

Study omitted	MD	95% CI	P-value	I^2	Tau^2
Omitting Su et al., 2020	0.13	[0.06; 0.19]	<0.001	50.1%	0.002
Omitting Gong et al., 2020	0.16	[0.08; 0.23]	<0.001	54.6%	0.004
Omitting Yao et al., 2022	0.15	[0.07; 0.22]	<0.001	62.0%	0.004
Omitting Yao et al., 2023	0.15	[0.09; 0.21]	<0.001	56.7%	0.003
Omitting Liu et al., 2025	0.12	[0.08; 0.16]	<0.001	13.7%	0.000
Omitting Cold et al., 2026	0.14	[0.07; 0.21]	<0.001	62.0%	0.003

Endoscopy | DOI 10.1055/a-2904-8262 | © The Author(s).

Fig. 10s PPC

(a) Forest plot comparing PPC between CAQ intervention and SC. The overall effect indicated that the use of CAQ significantly increased PPC by 0.32 polyps to SC (WMD: 0.31; 95% CI [0.17; 0.44], P<0.001). Given the substantial heterogeneity observed in this primary analysis (I^2 = 51.4%), a leave-one-out sensitivity analysis was conducted to identify sources of variance from individual studies. **(b)** Leave-one-out sensitivity analysis table. Each row demonstrated the recalculated metrics when the specified study was omitted from the meta-analysis. Noticeably, omitting Cold et al. reduced the heterogeneity remarkably (from I^2=51.4% to I^2=12.9%). Omission of a single study kept resulting in a significant WMD (range WMD: 0.13-0.53) favoring CAQ to SC.

Abbreviations: PPC = Polyps Per Colonoscopy; CAQ = Computer-aided Quality Assurance; CI = Confidence Interval; DL = DerSimonian-Laird; SC = Standard Colonoscopy; WMD = Weighted Mean Difference.

a

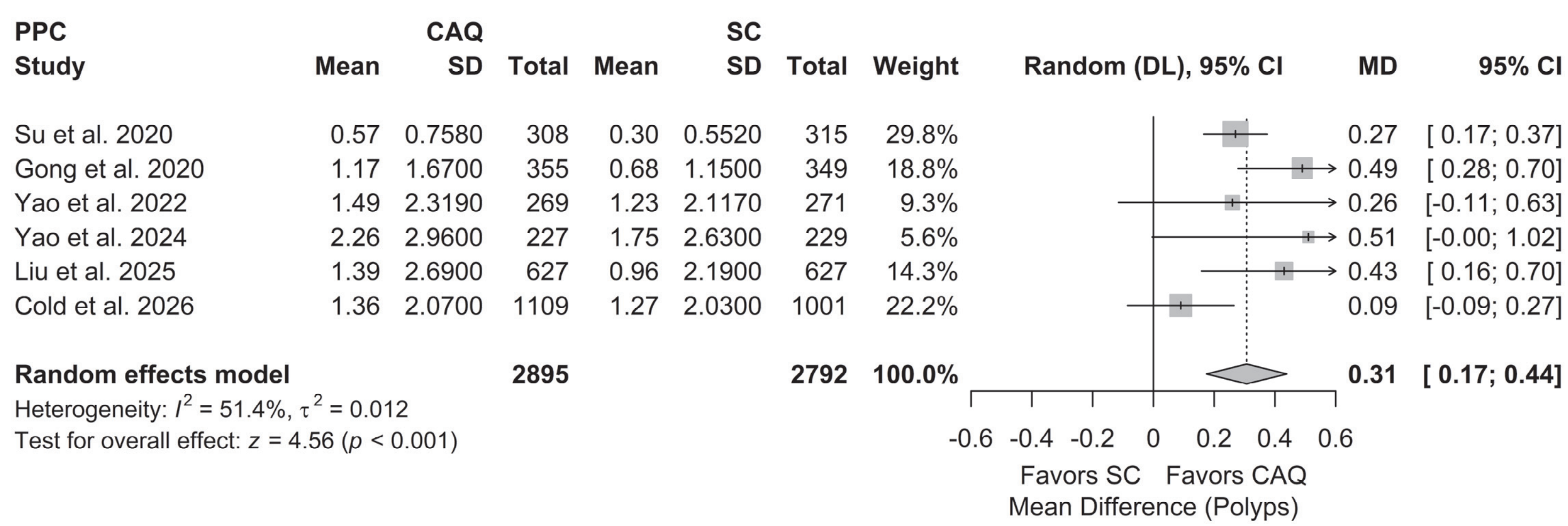

b

PPC Leave-one-out sensitivity analysis

Study omitted	MD	95% CI	P-value	I^2	Tau^2
Omitting Su et al., 2020	0.33	[0.14; 0.53]	<0.001	60.7%	0.028
Omitting Gong et al., 2020	0.26	[0.13; 0.38]	<0.001	33.6%	0.006
Omitting Yao et al., 2022	0.32	[0.17; 0.47]	<0.001	61.1%	0.016
Omitting Yao et al., 2023	0.30	[0.16; 0.43]	<0.001	58.0%	0.014
Omitting Liu et al., 2025	0.29	[0.14; 0.44]	<0.001	55.8%	0.014
Omitting Cold et al., 2026	0.34	[0.24; 0.45]	<0.001	12.9%	0.002

Endoscopy | DOI 10.1055/a-2904-8262 | © The Author(s).

Fig. 11s Withdrawal time

(a) Forest plot comparing withdrawal time between CAQ intervention and SC. The overall effect indicated that the use of CAQ significantly increased withdrawal time by 0.64 minutes (WMD: 0.64; 95% CI [0.04; 1.24], P=0.037). Given the substantial heterogeneity observed in this primary analysis (I^2 = 96.1%), a leave-one-out sensitivity analysis was conducted to identify sources of variance from individual studies. **(b)** Leave-one-out sensitivity analysis table. Each row demonstrated the recalculated metrics when the specified study was omitted from the meta-analysis. Noticeably, omitting Liu et al. reduced the heterogeneity remarkably (from I^2=96.1% to I^2=91.2%).

Abbreviations: CAQ = Computer-aided Quality Assurance; CI = Confidence Interval; DL = DerSimonian-Laird; SC = Standard Colonoscopy; WMD = Weighted Mean Difference.

a

Withdrawal time	CAQ			SC					
Study	Mean	SD	Total	Mean	SD	Total	Weight	MD	95% CI
Su et al. 2020	7.03	1.0100	308	5.68	1.2600	315	18.4%	1.35	[1.17; 1.53]
Gong et al. 2020	6.38	2.4800	355	4.76	2.5400	349	17.5%	1.62	[1.25; 1.99]
Yao et al. 2022	9.71	4.1300	269	10.14	4.1400	271	15.0%	-0.43	[-1.13; 0.27]
Yao et al. 2024	7.35	3.4100	227	7.32	2.9000	229	16.0%	0.03	[-0.55; 0.61]
Liu et al. 2025	6.78	1.1200	627	6.46	0.9700	627	18.6%	0.32	[0.20; 0.44]
Cold et al. 2026	16.00	8.5833	1109	15.25	8.9000	1001	14.5%	0.75	[0.00; 1.50]
**Random effects model**			**2895**			**2792**	**100.0%**	**0.64**	**[0.04; 1.24]**

Heterogeneity: I^2 = 96.1%, τ^2 = 0.502

Test for overall effect: z = 2.09 (p = 0.037)

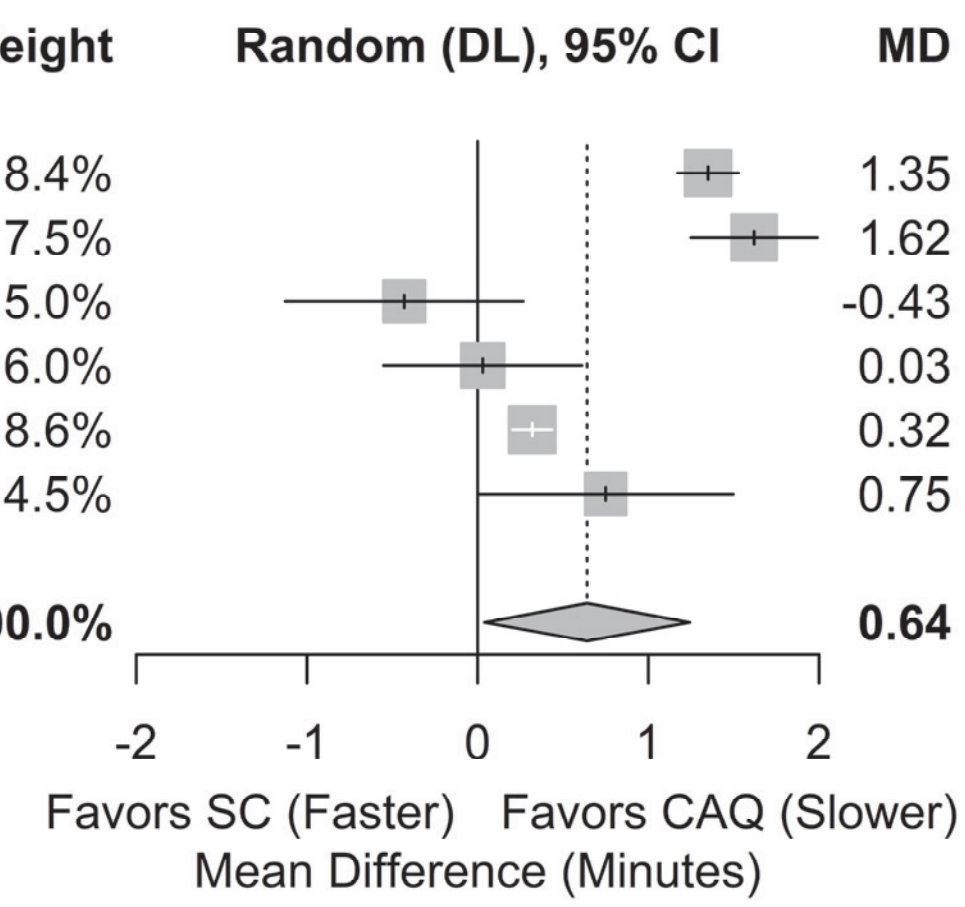

b

Leave-one-out meta-analysis (Withdrawal Time - DL)

Study omitted	MD	95% CI	P-value	I^2	Tau^2	Q-test P
Omitting Su et al., 2020	0.48	[-0.18; 1.14]	0.15	92.2%	0.49	<0.001
Omitting Gong et al., 2020	0.44	[-0.21; 1.08]	0.18	96.1%	0.47	<0.001
Omitting Yao et al., 2022	0.83	[0.19; 1.48]	0.012	96.7%	0.49	<0.001
Omitting Yao et al., 2023	0.76	[0.09; 1.42]	0.026	96.8%	0.52	<0.001
Omitting Liu et al., 2025	0.72	[0.06; 1.37]	0.033	91.2%	0.49	<0.001
Omitting Cold et al., 2026	0.62	[-0.04; 1.29]	0.07	96.9%	0.53	<0.001

Endoscopy | DOI 10.1055/a-2904-8262 | © The Author(s).

Fig. 12s Adenoma size

Forest plot comparing adenoma size between CAQ intervention and SC. The overall effect indicated that the use of CAQ significantly increased adenoma size ≤5 mm by 0.07 adenomas (WMD: 0.07; 95% CI [0.04; 0.10], P<0.001), adenomas 6-9 mm by 0.01 adenomas (WMD: 0.01; 95% CI [0.00; 0.0], P=0.005), and ≥10 mm by 0.03 adenomas (WMD: 0.03; 95% CI [0.02; 0.05], P<0.001). The effect of CAQ on adenoma detection varied significantly depending on lesion size (test for subgroup differences: I^2= 65.5%, P<0.001). Abbreviations: APC = Adenomas Per Colonoscopy; CAQ = Computer-aided Quality Assurance; CI = Confidence Interval; SC = Standard Colonoscopy; WMD = Weighted Mean Difference.

Adenoma Size

	CAQ			SC					
Study	Mean	SD	Total	Mean	SD	Total	Weight	MD	95% CI
**≤5 mm**									
Su et al. 2020	0.23	0.4835	308	0.12	0.3427	315	14.4%	0.12	[0.05; 0.18]
Gong et al. 2020	0.13	0.3600	355	0.07	0.2676	349	25.4%	0.06	[0.01; 0.10]
Yao et al. 2022	0.23	0.4762	269	0.14	0.3794	271	12.1%	0.08	[0.01; 0.16]
Yao et al. 2024	0.19	0.4301	227	0.18	0.4283	229	10.5%	0.00	[-0.08; 0.08]
Liu et al. 2025	0.26	0.5114	627	0.17	0.4150	627	21.8%	0.09	[0.04; 0.14]
Cold et al. 2026	0.56	0.7507	1109	0.51	0.7117	1001	15.8%	0.06	[-0.01; 0.12]
**Random effects model**			**2895**			**2792**	**100.0%**	**0.07**	**[0.04; 0.10]**
Heterogeneity: I^2 = 14.8%, τ^2 = 0.0002									
**6–9 mm**									
Gong et al. 2020	0.01	0.1061	355	0.00	0.0535	349	66.4%	0.01	[-0.00; 0.02]
Yao et al. 2022	0.04	0.2022	269	0.01	0.1215	271	12.8%	0.03	[-0.00; 0.05]
Yao et al. 2024	0.07	0.2737	227	0.05	0.2192	229	4.9%	0.03	[-0.02; 0.07]
Liu et al. 2025	0.11	0.3317	627	0.07	0.2709	627	9.1%	0.04	[0.00; 0.07]
Cold et al. 2026	0.21	0.4603	1109	0.20	0.4470	1001	6.8%	0.01	[-0.03; 0.05]
**Random effects model**			**2587**			**2477**	**100.0%**	**0.01**	**[0.00; 0.02]**
Heterogeneity: I^2 = 0%, τ^2 = 0									
**≥10 mm**									
Gong et al. 2020	0.03	0.1678	355	0.00	0.0535	349	27.7%	0.03	[0.01; 0.04]
Yao et al. 2022	0.03	0.1613	269	0.01	0.0859	271	23.8%	0.02	[-0.00; 0.04]
Yao et al. 2024	0.04	0.2099	227	0.02	0.1478	229	14.5%	0.02	[-0.01; 0.06]
Liu et al. 2025	0.06	0.2526	627	0.03	0.1830	627	21.2%	0.03	[0.01; 0.05]
Cold et al. 2026	0.22	0.4710	1109	0.15	0.3832	1001	12.8%	0.07	[0.04; 0.11]
**Random effects model**			**2587**			**2477**	**100.0%**	**0.03**	**[0.02; 0.05]**
Heterogeneity: I^2 = 45.1%, τ^2 = 0.0001									
Heterogeneity: I^2 = 56.6%, τ^2 = 0.0003									
Test for subgroup differences: χ^2_2 = 15.59, df = 2 (p = 0.0004)									

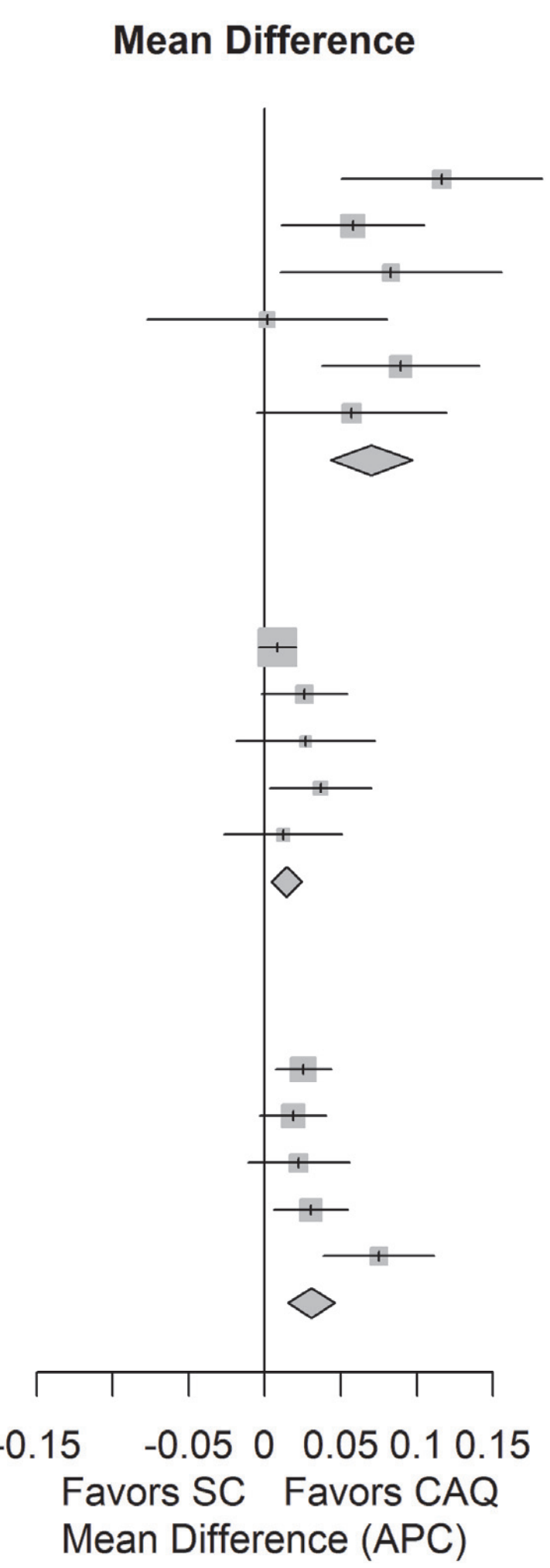

Endoscopy | DOI 10.1055/a-2904-8262 | © The Author(s).

Fig. 13s Leave-one-out sensitivity analysis adenoma size.

Leave-one-out analyses for adenoma size showed omission of most studies resulted in I^2=0.0%, except for adenomas ≥10 mm. These results should be interpreted with caution as the non-detectable heterogeneity may reflect limited statistical power due to the small number of included studies rather than definitive clinical homogeneity.

Leave-one-out Analysis: Adenoma Size

Category	Study Omitted	MD	95% CI	P-value	I^2	Tau^2
Size: <=5 mm	Su et al. 2020	-0.01	[-0.03; 0.00]	0.128	0.0	% 0.000
Size: <=5 mm	Gong et al. 2020	-0.01	[-0.02; 0.01]	0.221	0.0	% 0.000
Size: <=5 mm	Yao et al. 2022	-0.01	[-0.03; 0.00]	0.143	0.0	% 0.000
Size: <=5 mm	Yao et al. 2024	-0.01	[-0.03; 0.00]	0.167	0.0	% 0.000
Size: <=5 mm	Liu et al. 2025	-0.01	[-0.03; 0.01]	0.171	0.0	% 0.000
Size: <=5 mm	Cold et al. 2026	-0.01	[-0.04; 0.01]	0.182	0.0	% 0.000
Size: 6-9 mm	Gong et al. 2020	-0.00	[-0.01; 0.01]	0.764	0.0	% 0.000
Size: 6-9 mm	Yao et al. 2022	-0.00	[-0.01; 0.01]	0.875	0.0	% 0.000
Size: 6-9 mm	Yao et al. 2024	0.00	[-0.01; 0.01]	0.999	0.0	% 0.000
Size: 6-9 mm	Liu et al. 2025	0.00	[-0.01; 0.01]	0.621	6.2	% 0.000
Size: 6-9 mm	Cold et al. 2026	0.00	[-0.01; 0.02]	0.442	0.0	% 0.000
Size: >=10 mm	Gong et al. 2020	0.00	[-0.00; 0.01]	0.309	0.0	% 0.000
Size: >=10 mm	Yao et al. 2022	0.01	[-0.00; 0.02]	0.147	57.3	% 0.000
Size: >=10 mm	Yao et al. 2024	0.01	[-0.00; 0.02]	0.129	56.4	% 0.000
Size: >=10 mm	Liu et al. 2025	0.02	[0.00; 0.03]	0.049	50.7	% 0.000
Size: >=10 mm	Cold et al. 2026	0.02	[-0.00; 0.03]	0.074	57.2	% 0.000
OVERALL (ADR)	Su et al. 2020	0.07	[0.05; 0.10]	< 0.001	0.0	% 0.000
OVERALL (ADR)	Gong et al. 2020	0.08	[0.05; 0.11]	< 0.001	0.0	% 0.000
OVERALL (ADR)	Yao et al. 2022	0.08	[0.05; 0.11]	< 0.001	0.0	% 0.000
OVERALL (ADR)	Yao et al. 2024	0.08	[0.06; 0.11]	< 0.001	0.0	% 0.000
OVERALL (ADR)	Liu et al. 2025	0.07	[0.05; 0.10]	< 0.001	0.0	% 0.000
OVERALL (ADR)	Cold et al. 2026	0.09	[0.06; 0.12]	< 0.001	0.0	% 0.000

Endoscopy | DOI 10.1055/a-2904-8262 | © The Author(s).

Fig. 14s Adenoma location

Forest plot comparing adenoma location between CAQ intervention and SC. The overall effect indicated that the use of CAQ significantly increased adenoma location in ascending- by 0.03 adenomas (WMD: 0.03; 95% CI [0.02; 0.05], P<0.001), transverse- by 0.04 adenomas (WMD: 0.01; 95% CI [0.02; 0.05], P<0.001), descending- by 0.02 adenomas (WMD: 0.02; 95% CI [0.00; 0.03], P=0.015), sigmoid colon by 0.03 adenomas (WMD: 0.03; 95% CI [0.01; 0.05], P<0.001), and rectum by 0.01 adenomas (WMD: 0.01; 95% CI [0.00; 0.02], P=0.023), but not for cecum (WMD: 0.00; 95% CI [-0.00; 0.03].
Abbreviations: APC = Adenomas Per Colonoscopy; CAQ = Computer-aided Quality Assurance; CI = Confidence Interval; SC = Standard Colonoscopy; WMD = Weighted Mean Difference.

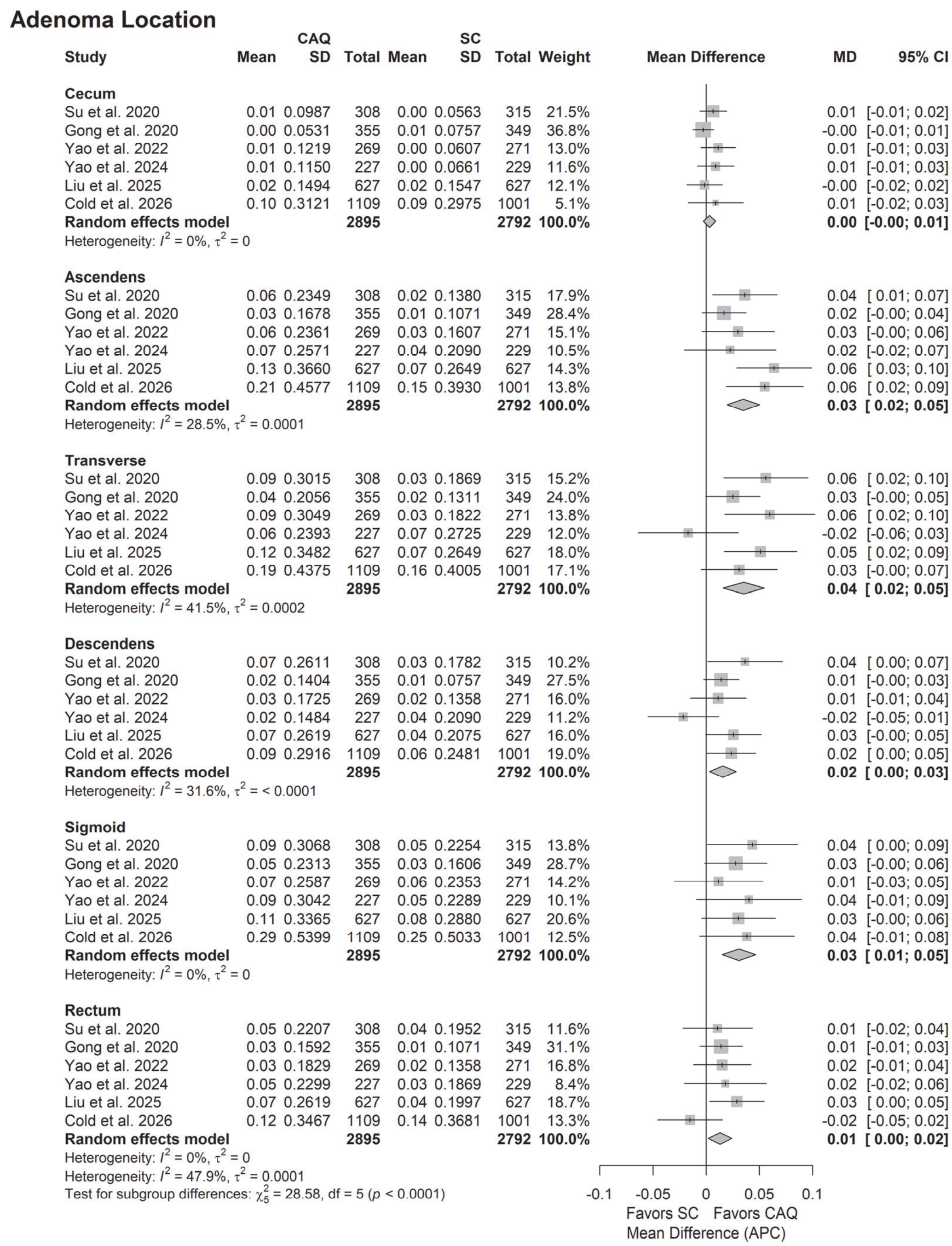

Endoscopy | DOI 10.1055/a-2904-8262 | © The Author(s).

Fig. 15s Leave-one-out sensitivity analyses for adenoma location

Leave-one-out analyses showed omission of most studies resulted in I^2=0.0%, except for ascending, transverse-, and descending colon and rectum. These results should be interpreted with caution as the non-detectable heterogeneity may reflect limited statistical power due to the small number of included studies rather than definitive clinical homogeneity.

Leave-one-out Analysis: Adenoma Location (Mean Difference)

Location	Study Omitted	MD	95% CI	P-value	I^2	Tau^2	Q-test P
Cecum	Su et al. 2020	0.00	[-0.00; 0.01]	0.530	0.0 %	0.000	0.515
Cecum	Gong et al. 2020	0.01	[-0.00; 0.01]	0.082	0.0 %	0.000	0.860
Cecum	Yao et al. 2022	0.00	[-0.00; 0.01]	0.561	0.0 %	0.000	0.638
Cecum	Yao et al. 2024	0.00	[-0.00; 0.01]	0.467	0.0 %	0.000	0.533
Cecum	Liu et al. 2025	0.00	[-0.00; 0.01]	0.244	0.0 %	0.000	0.509
Cecum	Cold et al. 2026	0.00	[-0.00; 0.01]	0.368	0.0 %	0.000	0.488
Ascendens	Su et al. 2020	0.04	[0.02; 0.05]	< 0.001	42.4 %	0.000	0.139
Ascendens	Gong et al. 2020	0.04	[0.03; 0.06]	< 0.001	0.0 %	0.000	0.497
Ascendens	Yao et al. 2022	0.04	[0.02; 0.06]	< 0.001	42.6 %	0.000	0.138
Ascendens	Yao et al. 2024	0.04	[0.02; 0.05]	< 0.001	40.8 %	0.000	0.150
Ascendens	Liu et al. 2025	0.03	[0.01; 0.04]	< 0.001	0.0 %	0.000	0.460
Ascendens	Cold et al. 2026	0.03	[0.02; 0.05]	< 0.001	25.4 %	0.000	0.252
Transverse	Su et al. 2020	0.03	[0.01; 0.05]	0.004	45.0 %	0.000	0.122
Transverse	Gong et al. 2020	0.04	[0.01; 0.06]	0.002	48.2 %	0.000	0.102
Transverse	Yao et al. 2022	0.03	[0.01; 0.05]	0.003	43.3 %	0.000	0.133
Transverse	Yao et al. 2024	0.04	[0.02; 0.06]	< 0.001	0.0 %	0.000	0.489
Transverse	Liu et al. 2025	0.03	[0.01; 0.05]	0.006	46.7 %	0.000	0.112
Transverse	Cold et al. 2026	0.04	[0.01; 0.06]	0.003	52.9 %	0.000	0.075
Descendens	Su et al. 2020	0.01	[0.00; 0.03]	0.046	31.7 %	0.000	0.210
Descendens	Gong et al. 2020	0.02	[-0.00; 0.03]	0.068	44.8 %	0.000	0.123
Descendens	Yao et al. 2022	0.02	[0.00; 0.03]	0.037	44.4 %	0.000	0.126
Descendens	Yao et al. 2024	0.02	[0.01; 0.03]	< 0.001	0.0 %	0.000	0.734
Descendens	Liu et al. 2025	0.01	[-0.00; 0.03]	0.071	40.0 %	0.000	0.154
Descendens	Cold et al. 2026	0.01	[-0.00; 0.03]	0.080	40.9 %	0.000	0.149
Sigmoid	Su et al. 2020	0.03	[0.01; 0.05]	< 0.001	0.0 %	0.000	0.904
Sigmoid	Gong et al. 2020	0.03	[0.01; 0.05]	< 0.001	0.0 %	0.000	0.847
Sigmoid	Yao et al. 2022	0.03	[0.02; 0.05]	< 0.001	0.0 %	0.000	0.974
Sigmoid	Yao et al. 2024	0.03	[0.01; 0.05]	< 0.001	0.0 %	0.000	0.864
Sigmoid	Liu et al. 2025	0.03	[0.01; 0.05]	< 0.001	0.0 %	0.000	0.838
Sigmoid	Cold et al. 2026	0.03	[0.01; 0.05]	< 0.001	0.0 %	0.000	0.859
Rectum	Su et al. 2020	0.01	[-0.00; 0.03]	0.053	16.6 %	0.000	0.309
Rectum	Gong et al. 2020	0.01	[-0.00; 0.03]	0.106	16.7 %	0.000	0.308
Rectum	Yao et al. 2022	0.01	[-0.00; 0.03]	0.079	16.5 %	0.000	0.310
Rectum	Yao et al. 2024	0.01	[-0.00; 0.03]	0.063	15.7 %	0.000	0.314
Rectum	Liu et al. 2025	0.01	[-0.00; 0.02]	0.141	0.0 %	0.000	0.550
Rectum	Cold et al. 2026	0.02	[0.01; 0.03]	0.005	0.0 %	0.000	0.902
OVERALL (ADR)	Su et al. 2020	0.07	[0.05; 0.10]	< 0.001	0.0 %	0.000	0.714
OVERALL (ADR)	Gong et al. 2020	0.08	[0.05; 0.11]	< 0.001	0.0 %	0.000	0.468
OVERALL (ADR)	Yao et al. 2022	0.08	[0.05; 0.11]	< 0.001	0.0 %	0.000	0.482
OVERALL (ADR)	Yao et al. 2024	0.08	[0.06; 0.11]	< 0.001	0.0 %	0.000	0.588
OVERALL (ADR)	Liu et al. 2025	0.07	[0.05; 0.10]	< 0.001	0.0 %	0.000	0.552
OVERALL (ADR)	Cold et al. 2026	0.09	[0.06; 0.12]	< 0.001	0.0 %	0.000	0.640

Endoscopy | DOI 10.1055/a-2904-8262 | © The Author(s).
